# Hdac1 and Hdac2 regulate the quiescent state and survival of hair-follicle mesenchymal niche

**DOI:** 10.1038/s41467-023-40573-7

**Published:** 2023-08-10

**Authors:** Hadas Sibony-Benyamini, Emil Aamar, David Enshell-Seijffers

**Affiliations:** The Laboratory of Developmental Biology, The Azrieli Faculty of Medicine, Bar Ilan University, 8 Henrietta Szold, Safed, Israel

**Keywords:** Stem-cell niche, Regeneration, Post-translational modifications, Histone post-translational modifications

## Abstract

While cell division is essential for self-renewal and differentiation of stem cells and progenitors, dormancy is required to maintain the structure and function of the stem-cell niche. Here we use the hair follicle to show that during growth, the mesenchymal niche of the hair follicle, the dermal papilla (DP), is maintained quiescent by the activity of Hdac1 and Hdac2 in the DP that suppresses the expression of cell-cycle genes. Furthermore, Hdac1 and Hdac2 in the DP promote the survival of DP cells throughout the hair cycle. While during growth and regression this includes downregulation of p53 activity and the control of p53-independent programs, during quiescence, this predominantly involves p53-independent mechanisms. Remarkably, Hdac1 and Hdac2 in the DP during the growth phase also participate in orchestrating the hair cycle clock by maintaining physiological levels of Wnt signaling in the vicinity of the DP. Our findings not only provide insight into the molecular mechanism that sustains the function of the stem-cell niche in a persistently changing microenvironment, but also unveil that the same mechanism provides a molecular toolbox allowing the DP to affect and fine tune the microenvironment.

## Abstract (web summary)

Cell division and stem cell maintenance are tightly linked. Here they show that in the hair follicle an epigenetic mechanism maintains mesenchymal niche dormancy in a highly proliferative microenvironment while repurposing mitogenic signaling to orchestrate the hair cycle clock.

## Introduction

The intrinsic feature of stem cells to self-renew and concomitantly to generate progenitors that are committed to undergo differentiation requires the presence of mitogenic signals that are provided by the microenvironment, often called the niche. Remarkably, the cells that constitute the niche often remain quiescent despite their exposure to the same proliferation cues. While the molecular mechanisms by which the niche regulates stem-cell activity and progenitor behavior are under intensive study^1^, the mechanisms that allow the cellular constituents of the niche to ignore the mitogenic microenvironment and remain dormant are poorly understood. The hair follicle is a powerful model system to interrogate stem-cell niche dormancy due to its accessibility, its well-characterized stem cells and progenitors, and its well-defined anatomy^2,3^.

Hair follicles undergo cycles of growth (anagen), destruction (catagen), quiescence (telogen) and regeneration^4–6^. In each phase of the hair cycle, follicles adopt a different morphology and structure, and therefore, the transitions between these phases involve dramatic morphological alterations. This metamorphosis of the follicle during the hair cycle requires not only the exquisite coordination of multiple biological pathways but also entails the presence of epithelial stem and progenitor cell populations. Therefore, the hair follicle provides a unique microenvironment that sustains stem-cell identity while allows dividing progeny to “migrate” down the stemness hierarchy to become progressively lineage-restricted and consequently proceed through differentiation programs. The niche of the hair follicle is composed of multiple components that some are constantly rearranged as a result of the morphological changes imposed by the hair cycle. This constant rearrangement of the niche further entails additional level of regulation that maintains the functionality of the niche despite its structural redisposition during the hair cycle.

The mature hair follicle during the hair cycle is largely composed of two compartments derived from embryonically distinct origins; the follicular epidermis with its differentiated and undifferentiated epithelial cell types, collectively called keratinocytes, and the dermal papilla (DP), a compact group of mesenchymal cells at the base of the follicle that constitutes an important part of the progenitor and stem-cell niche and plays an important role in regulating different aspects of hair biology^7^. During anagen, differentiated keratinocytes within the distal part of the follicle are arranged according to their lineage identity in 8 concentric layers that form a cylinder with three domains: the outer root sheath (ORS), inner root sheath (IRS) and hair shaft (HS) that include one, four and three layers, respectively^8^. The lineage identity of the layers in the IRS and HS arises in the proximal part of the follicle, the bulb region, that includes the embedded DP and the surrounding, highly proliferative, matrix cells. Matrix cells in close contact with the DP function as progenitors; they undergo asymmetric division to renew the matrix progenitor pool abutting the DP and to generate transit-amplifying progeny that are displaced away from the DP^9^. The transit-amplifying progeny moves upwards while they undergo a limited number of divisions and subsequently differentiate along a largely specific lineage and according to the position of the ancestor around the DP^9–11^. The orchestrated and coordinated self-renewal and differentiation of the matrix progenitor cells allow hair formation, growth, and emergence.

Anagen ends when proliferation in the matrix ceases, and catagen is initiated when matrix cells around the DP apoptose^12,13^. Apoptosis spreads from the matrix upwards while the hair shaft slides towards the upper part of the follicle. Consequently, the follicle shrinks and regresses while the DP, surviving the apoptotic environment, is withdrawn with the regressing epithelial strand. Following the destruction of the bottom two thirds of the follicle, the upper ORS collapses around the bottom end of the hair to form the bulge region and the secondary germ^14,15^. Catagen ends when the DP completes its relocation to lie beneath the secondary germ and when the bottom end of the hair becomes anchored within the bulge. A quiescent phase of variable length precedes anagen induction of the next cycle by the DP^16,17^. At the onset of a new anagen phase, primed stem cells in the secondary germ adjacent to the DP proliferate to form a new pool of matrix progenitor cells and to regenerate the inner layers of the lower follicle. Subsequently, quiescent stem cells in the bulge region are activated for a short period during the regeneration of the follicle to restore the ORS and to produce the ancestors that will form the new bulge and secondary germ at the end of the cycle^18^.

While the mechanism that drives the cyclic migration of the DP between the upper and lower dermis is beginning to unravel^19^, the mechanism that maintains the DP integrity and functionality during these morphological transformations of the hair cycle is poorly understood. Previous reports indicated that while the number of DP cells fluctuates to some extent during the hair cycle, very little proliferation or apoptosis was observed in the DP despite the highly mitogenic and apoptotic microenvironments the DP resides in and passes through during the hair cycle, respectively^20,21^. We hypothesized that epigenetic mechanisms operate within the DP to retain niche quiescence and survival, and in the current study, we focused on exploring the role of histone deacetylation in regulating the DP function during the hair cycle.

Histone acetylation and deacetylation serves as a key modulator of nucleosomal structure that directly regulates transcription and therefore provides an efficient platform to couple gene expression with extracellular signaling. Two large families of enzymes control the acetylation state of the chromatin; histone acetyltransferases (HATs) and histone deacetylases (HDACs)^22–24^. The balance between the antagonistic actions of these enzymes provides one mechanism to govern gene transcription. Furthermore, the ability of these enzymes to modify and control the function of non-histone proteins broadens their mechanistic action in this regulation. In mammals, HDACs comprise a large family of 18 deacetylases that have been classified into 4 classes based on sequence similarity^22^. Class I, II, and IV represent the classic HDACs while class III consists of NAD-dependent functionally unrelated deacetylases called sirtuins. The subfamily with the most definitive role in regulating gene expression and shows the strongest histone deacetylase activity are the class I HDACs: 1, 2, 3 and 8. Hdac1 and Hdac2 are nearly identical (83% identity) and thought to originate from a gene duplication event in the common ancestor of all vertebrates^23,24^. It is therefore unsurprising that Hdac1 and Hdac2 are functionally redundant, although they do perform non-redundant roles in early development.

Concomitant ablation of *Hdac1* and *Hdac2* in the epidermis before follicle formation revealed the important role these histone-modifying enzymes play in mediating the repressive function of p63 during epidermis formation in the embryo^25^. While the role of these enzymes in the DP has not been studied in this previous work, the immunostaining analysis performed in that study clearly showed that both Hdac1 and Hdac2 are expressed in the DP^25^. Here, we show that Hdac1 and Hdac2 are both required to maintain the quiescent state and survival of DP cells. Dormancy of DP cells is conferred by the activity of Hdac1 and Hdac2 that suppresses the expression of cell-cycle genes such as CycD1, preventing DP cells to respond to the mitogenic signals abundantly present in the vicinity of the DP during anagen. Concomitant ablation of *Hdac1* and *Hdac2* specifically in the DP lifts this inhibition, unleashing the DP to proliferate only during anagen. Moreover, we find that *Hdac1* and *Hdac2* abrogation in the DP also results in p53-dependent and independent apoptosis of DP cells. While DP apoptosis during anagen is counterbalanced by proliferation and thus the DP role in regulating the production of hair shafts is maintained, the progressive loss of DP cells during telogen without replenishment leads to telogen arrest. Furthermore, the DP maintenance activity of Hdac1 and Hdac2 is coupled to the role of the DP in orchestrating the hair-cycle clock by actively sustaining the physiological levels of Wnt signaling in the vicinity of the DP. In the absence of Hdac1 and Hdac2 in the DP, Wnt signaling in both the matrix and DP is reduced and the anagen phase shortens. Together our findings unravel the multi-modal action of Hdac1 and Hdac2 in preserving the function of the DP and thus, enabling the DP to cope with the persistently changing microenvironment required to execute the hair cycle.

## Results

### Concomitant ablation of *Hdac1* and *Hdac2* specifically in the DP results in progressive hair phenotype

**Fig. 1:**
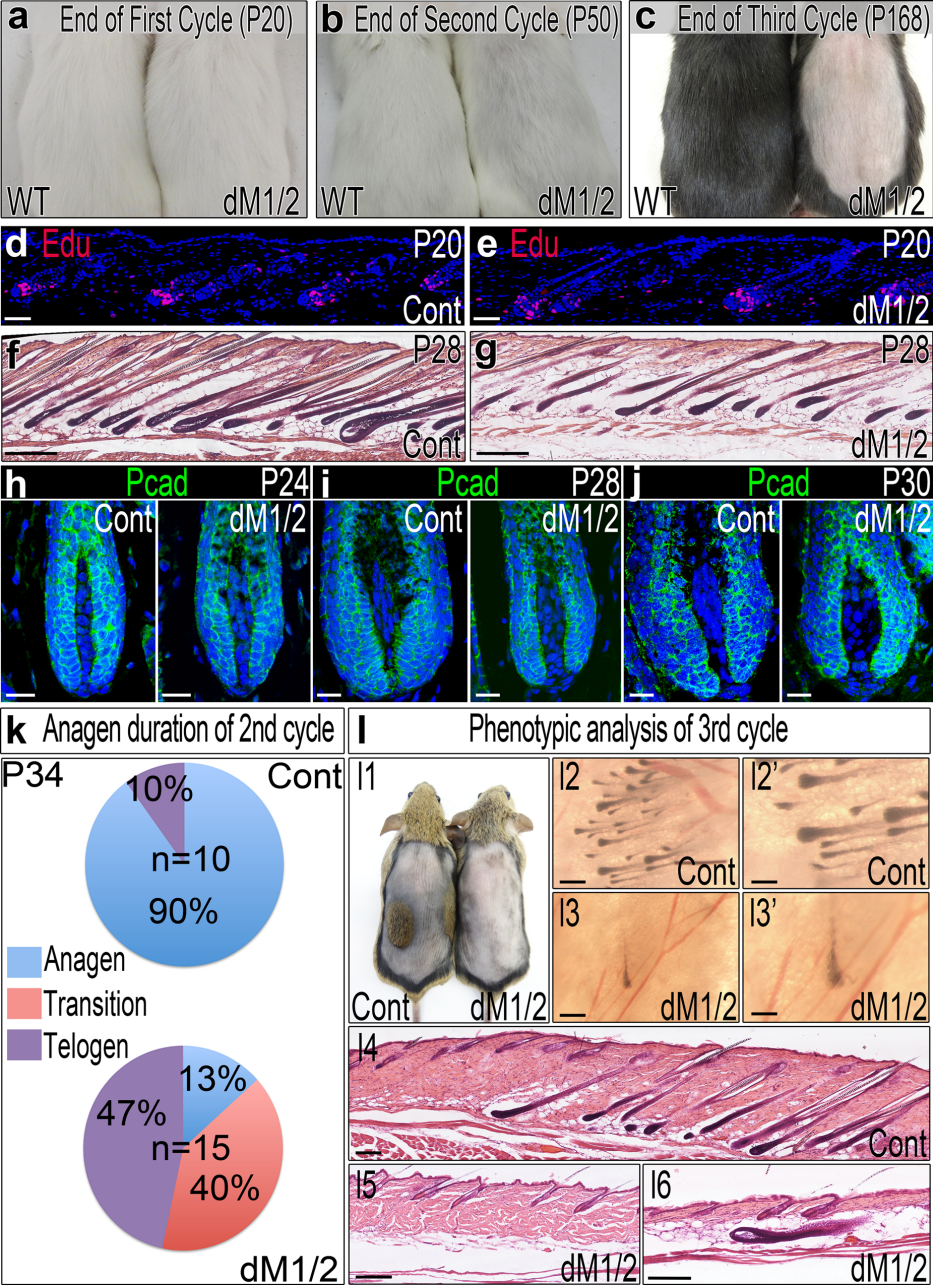
Ablation of Hdac1 and Hdac2 in the DP results in progressive hair phenotype. **a**–**c** Images of wild-type (WT) and double mutant (dM1/2) mice at the end of the first, second and third hair cycle are shown. Note that in **a** and **b**, white mice are shown to easily demonstrate the abnormal hair coat generated after the second cycle in the double mutant. In **c**, black mice are shown to readily illustrate the failure to grow new hair coat in the double mutant after the third cycle. **d**, **e** Edu incorporation at P20 2 h post injection to demonstrate proliferation within the secondary germ, a first sign of anagen induction. *n* > 8 mice per genotype. Scale bar; 50 μm. **f**, **g** HE staining of skin sections at mid-anagen of the second cycle (P28). *n* > 10 mice per genotype. Scale bar; 250 μm. **h**–**j** Immunostaining for Pcad is shown to outline the DP and to illustrate the alterations in bulb structure and shape. *n* > 5 mice per genotype per stage. Scale bar; 20 μm. **k** Pie histogram summarizes the hair-cycle phase distribution of 10 control and 15 double mutant mice at P34. Transition includes skins at catagen or skins with an anterior-posterior gradient between phases (anterior at catagen while posterior at anagen, or anterior at telogen and posterior at catagen). **l1** The hair coat of control and mutant mice was clipped at the end of the second cycle (P50), and regrowth of the hair coat of the third cycle was followed. The image was taken at P115. **l2**, **l2’** A control skin viewed from the dermis side showing a small patch of hair follicles in anagen. L2’ is higher magnification of L2. **l3**, **l3’** A double mutant skin viewed from the dermis side showing a single follicle in anagen. **l4** HE staining shows a control skin section within the growing patch and its surroundings. **l5**, **l6** HE staining of a double mutant skin showing two adjacent sections. *n* > 14 mice per genotype. Scale bar in l2–l6; 200 μm. Scale bar in l2’–l3’; 100 μm. Source data are provided as a Source Data file.

To determine whether Hdac1 and Hdac2 play a role in the DP in regulating the hair cycle, *Hdac1* and *Hdac2* were ablated specifically in the DP postnatally. For this, the Corin-Cre mouse line^26^ was crossed with mouse lines that harbor conditional alleles of *Hdac1* and *Hdac2*^27^. Crosses were designed to obtain controls, single *Hdac1*, single *Hdac2*, and double *Hdac1/2* mutants. To uncover the outcomes of *Hdac* ablation in the DP, controls and mutants were phenotypically analyzed as follows (Supplementary Fig. 1a–c). Mice were observed for hair phenotype at P20 when follicles are known to be at the end of the first hair cycle (Supplementary Fig. 1a). At P20, the hair coat was removed by clipping, and the growth of the new hair coat was followed to the end of the second cycle around P50 when follicles have already entered the long telogen (Supplementary Fig. 1b). At P50, the hair coat was clipped again and the emergence of the new hair coat of the third cycle was documented (Supplementary Fig. 1c). No hair phenotype was observed in both single *Hdac1* and single *Hdac2* mutants throughout all three cycles. Furthermore, mice which are homozygous for mutant *Hdac1* and heterozygous for *Hdac2* and vice versa also lack a hair phenotype. In contrast, double *Hdac1/2* mutant mice (designated dM1/2) exhibit a progressive hair phenotype (Fig. 1a–c; Supplementary Fig. 1a–c). While hair length, structure, and type-frequency remain unaltered and the hair coat appears completely normal following the first cycle (Fig. 1a; Supplementary Fig. 1a, d, e), hairs of the second cycle are shorter and thinner, resulting in abnormal appearance of the second-cycle hair coat (Fig. 1b; Supplementary Fig. 1b, e, f). Furthermore, the hair coat of the double mutant fails to grow during the third cycle (Fig. 1c; Supplementary Fig. 1c).

### Hdac1 and Hdac2 in the DP are dispensable during the mid-anagen of the first cycle

Note that ablation of *Hdac1* and *Hdac2* in the DP results in distinct phenotypic outcomes for each cycle. Furthermore, the severity of the phenotype progressively increases from cycle to cycle. To evaluate whether the lack of phenotypic alterations at the end of the first cycle and the increase in phenotypic severity in subsequent cycles are a result of slow and progressive deletion of the floxed alleles or whether phenotypic progressiveness reflects functional complexity of Hdac1/2 in the DP, deletion efficiency was scored by using anti-Hdac1 and anti-Hdac2 antibodies to immunostain skin sections derived from dM1/2 mutant and control mice at mid-anagen of the first cycle when Cre activity is maximal^26^ (Supplementary Fig. 2a–e). Both Hdac1 and Hdac2 were ubiquitously detected in the nuclei of all cell types of control skin including the DP, consistent with previous reports^25^. In contrast, only a few DP cells positive for Hdac1 and Hdac2 are found in the DP of mutant mice whereas staining outside the DP remains unaltered, illustrating that deletion of *Hdac1* and *Hdac2* in the DP is already efficient during the mid-anagen of the first cycle. Note that while about 20% of DP cells (which is 4–6 DP cells per follicle) still express *Hdac1/2* (Supplementary Fig. 2e), this is far below the minimal 10 DP cells required for functional DP^28^. To further investigate whether the absence of Hdac1 and Hdac2 in the DP during the first cycle may still have minor effects on hair formation and differentiation, the expression patterns and levels of trichohyalin, hair keratins and Gata3 (all involved in the differentiation of the inner layers of the hair follicle) during the anagen of the first cycle were assessed by immunostaining and found unaltered in the mutant (Supplementary Fig. 1g), in line with the lack of hair phenotype.

To further explore the biological relevance of the lack of hair phenotype at the first cycle despite efficient deletion of *Hdac1* and *Hdac2*, the acetylation state of DP chromatin was addressed by immunostaining with an antibody that recognizes the acetylated form of K9 of Histone 3 (anti-H3K9Ac) during mid to late anagen of the first and second cycles (Supplementary Fig. 3a, b). As this acetylation mark often occurs in all cell types, the antibody was serially diluted to achieve a concentration that detects low levels of chromatin acetylation in wild-type DP cells. While this analysis revealed a slight increase of chromatin acetylation in mutant DP cells, this small incline of chromatin acetylation in the DP is comparable between first and second cycles, refuting the proposal that a time gap between deletion and chromatin acetylation in the DP is the underlying mechanism for the lack of hair phenotype during the first cycle. Together, these data suggest that the function of Hdac1 and Hdac2 in the DP is limited to a specific time window during the anagen of the second cycle and that both Hdac1 and Hdac2 in the DP are dispensable during mid-anagen of the first cycle.

### Hdac1 and Hdac2 in the DP regulate bulb morphology and anagen duration during the second cycle

The slight increase of chromatin acetylation in the mutant DP during mid to late anagen of the first cycle presumably alters the DP transcriptome to some extent. While these alterations obviously do not culminate into phenotypic effects, they may paracrinally change the attributes of the stem cells and their niche in the bulge and/or secondary germ compartments during telogen when the DP is in close proximity to these compartments, and consequently, affect the second hair cycle. Immunostaining analysis of structural and stem-cell markers such as Sox9, Nfatc1, CD34, K15, K6, K14 and Pcad revealed no alterations in the expression patterns or levels of these markers (Supplementary Fig. 1h), suggesting that stem-cell properties and niche structure remain unaltered in the mutant during first-cycle telogen. Furthermore, Edu labeling to assess anagen induction of the second cycle revealed that anagen induction occurs on time (Fig. 1d, e). Subsequently, regeneration proceeds normally despite the absence of Hdac1 and Hdac2 in the DP (Supplementary Fig. 2f, g). However, the regenerated mutant follicles are thinner than controls (Fig. 1f, g). Closer inspection of the follicles exposed morphological alterations in the bulb region of mutant mice (Fig. 1h–j; Supplementary Fig. 4a). At P24, following regeneration, the morphology of the bulb region in the mutant is similar to controls (Fig. 1h). Note the rounded shape and the unordered position of DP nuclei in both control and mutant mice. At P28, DP nuclei in controls acquire an elliptical shape and become tightly organized in parallel to the distal-proximal axis of the DP (Fig. 1i; Supplementary Fig. 4a). In contrast, DP nuclei in the mutant remain relatively rounded and unordered, and persist in this loose configuration throughout the anagen phase of the second cycle (Fig. 1j). Furthermore, while the matrix in controls increases in size and tightly envelopes the DP leaving a very narrow canal that bridges the DP to the dermis, the increase in mutant matrix is very little and the DP canal remains wide open. Remarkably, the lack of increase in matrix volume of the mutant is not due to reduced proliferation rate. Both Edu labeling with very short chase (15–30 min) and phospho-Histone 3 (pH3) immunostaining failed to detect significant alterations in the rate of proliferation within the matrix (Supplementary Fig. 4b–e). In addition, monitoring the transition from anagen through catagen to telogen of the second cycle revealed that anagen duration is substantially shorter in the mutant (Fig. 1k; Supplementary Fig. 1i, j), consistent with shorter hair shafts **(**Supplementary Fig. 1f, right histogram).

### Ablation of *Hdac1* and *Hdac2* in the DP results in telogen arrest following the second cycle

To explore whether the failure to regrow hair coat during the third cycle is due to telogen arrest, telogen delay, or abnormal anagen with aborted hair production, control and mutant mice were clipped and followed for months from the mid telogen of the second cycle. Note that telogen duration of the second cycle substantially varies and lasts from weeks to months in wild-type mice. Furthermore, anagen induction of the third cycle is highly unsynchronized and occurs in patches due to the concomitant presence of inhibitory and permissive macroenvironments^29^. Following few weeks, patches of hair regrowth were observed in control mice (Fig. 1l1). In contrast, no patches of hair regrowth were observed in dM1/2 mutant mice. However, single, unclipped, new isolated hairs were detected in the skin of the double mutant (Supplementary Fig. 2h, h**’**). Close inspection of control and mutant skins from the dermis side clearly demonstrated the presence of single anagen follicles in the mutant (Fig. 1l2, l3), suggesting that the isolated unclipped hairs are the product of follicles that enter a new cycle and therefore may represent escapers with inefficient deletion. Indeed, immunostaining analysis with anti-Hdac1 and anti-Hdac2 revealed inefficient ablation of Hdac1 and Hdac2 in the DP of those escapers (Supplementary Fig. 2i). While the lack of hair growth in the mutant and the unsynchronized nature of the third cycle prevent the distinction between permissive and inhibitory skin regions and thus complicate the analysis, the presence of these escapers in the mutant indicates a domain in which the macroenvironment is no longer inhibitory and thus allows anagen induction. Therefore, the presence of telogen follicles in such anagen-promoting regions suggests that these follicles are arrested in telogen, and indeed, serial section analysis around these anagen escapers revealed that all the surrounding follicles are morphologically in telogen (Fig. 1l4–6).

### Hdac1 and Hdac2 regulate transcriptional activity in the DP predominantly during early to mid-anagen of the second cycle

**Fig. 2:**
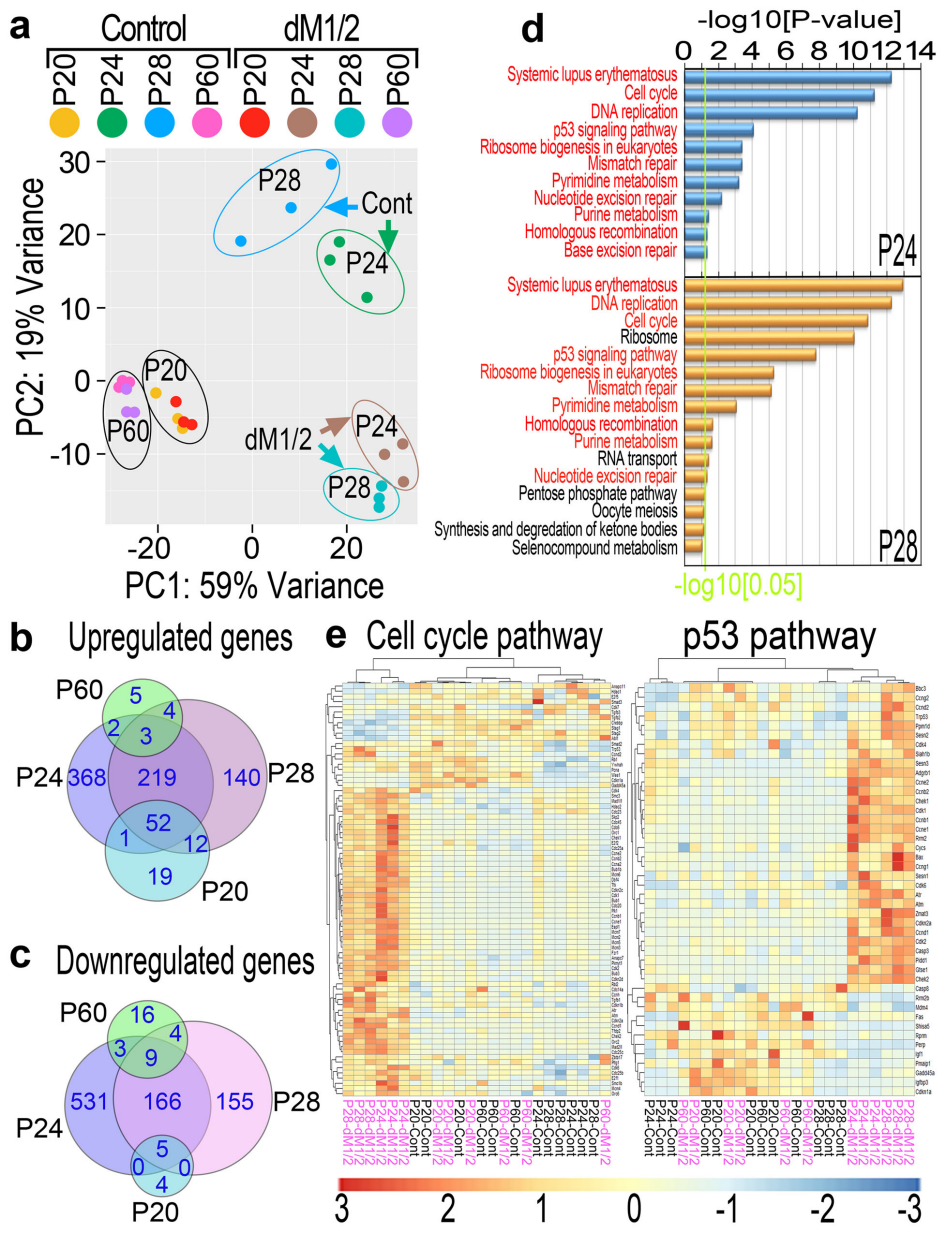
Gene expression profiling of DP cells revealed elevation of cell cycle and p53 pathways in dM1/2 mutant DP specifically during anagen. **a** A principle component analysis. **b**, **c** Venn diagram of upregulated (**b**) and downregulated (**c**) genes in the dM1/2 mutant vs. control. Note the genes altered during the telogen of the first cycle (P20) are distinct than those altered during the telogen of the second cycle (P60). **d** KEGG analysis of significantly altered pathways. Only pathways that are upregulated specifically during anagen are shown. Pathways in red are altered in both early (P24) and mid (P28) anagen. The green line marks *P*-value of 0.05. **e** Hierarchal clustering analysis of genes in the cell cycle (left) and p53 (right) pathways whose expression is altered in the dM1/2 mutant. Note in both pathways, mutant mice in anagen (P24 and P28) cluster together. Mutant and control mice are indicated in pink and black, respectively.

To unveil the molecular mechanism underlying this progressive phenotype, gene expression profiling of DP cells, isolated from control and mutant mice during telogen of the first (P20) and second (P60) cycles as well as during early (P24) and mid (P28) anagen of the second cycle, was performed by RNA-sequencing (RNA-seq) analysis. For this, DP cells were endogenously labeled with YFP and FACS-sorted as we have previously reported^30,31^. Principle component analysis illustrated that while the DP transcriptome during both the first (P20) and second (P60) telogen undergoes very little alterations, the DP transcriptome during both early (P24) and mid (P28) anagen is dramatically changed in the mutant (Fig. 2a), suggesting that Hdac1 and Hdac2 play an important role in the DP predominantly during anagen of the second cycle. Furthermore, the DP transcriptome of control mice at early anagen (P24) differs substantially from that of control mice at mid-anagen (P28), suggesting that following regeneration the DP in wild-type mice normally undergoes further transcriptional modifications that are required for DP function. In contrast, the DP transcriptome of mutant mice undergoes little change from early (P24) to mid (P28) anagen (Fig. 2a). This transcriptomics analysis correlates with the alterations in bulb morphology that normally occur immediately post regeneration (Fig. 1h–j). At P24, the bulb morphology and size of control and mutant mice are similar. However, while the matrix of control mice increases in size from P24 to P28, the matrix size of mutant mice at P28 resembles the one at P24, implying that transcriptional alterations in DP cells after regeneration is required to achieve proper matrix size. Venn analysis further corroborated the dramatic alterations in mutant DP transcriptome during anagen and the very moderate alterations during telogen (Fig. 2b, c). Furthermore, KEGG analysis of the RNA-seq data unraveled numerous biological pathways that are significantly altered in the mutant only during anagen (Fig. 2d). Remarkably, both p53 and the cell-cycle pathways are altered in mutant DP, and hierarchical clustering analysis of the genes altered in these pathways revealed that elevated expression of many of these genes is predominantly observed in the mutant during anagen (Fig. 2e). This analysis suggests that in the absence of Hdac1 and Hdac2, DP cells undergo both proliferation and apoptosis, processes that are rarely observed in normal DPs.

### Hdac1 and Hdac2 protect DP cells from apoptosis during anagen by suppressing p53 activity

**Fig. 3:**
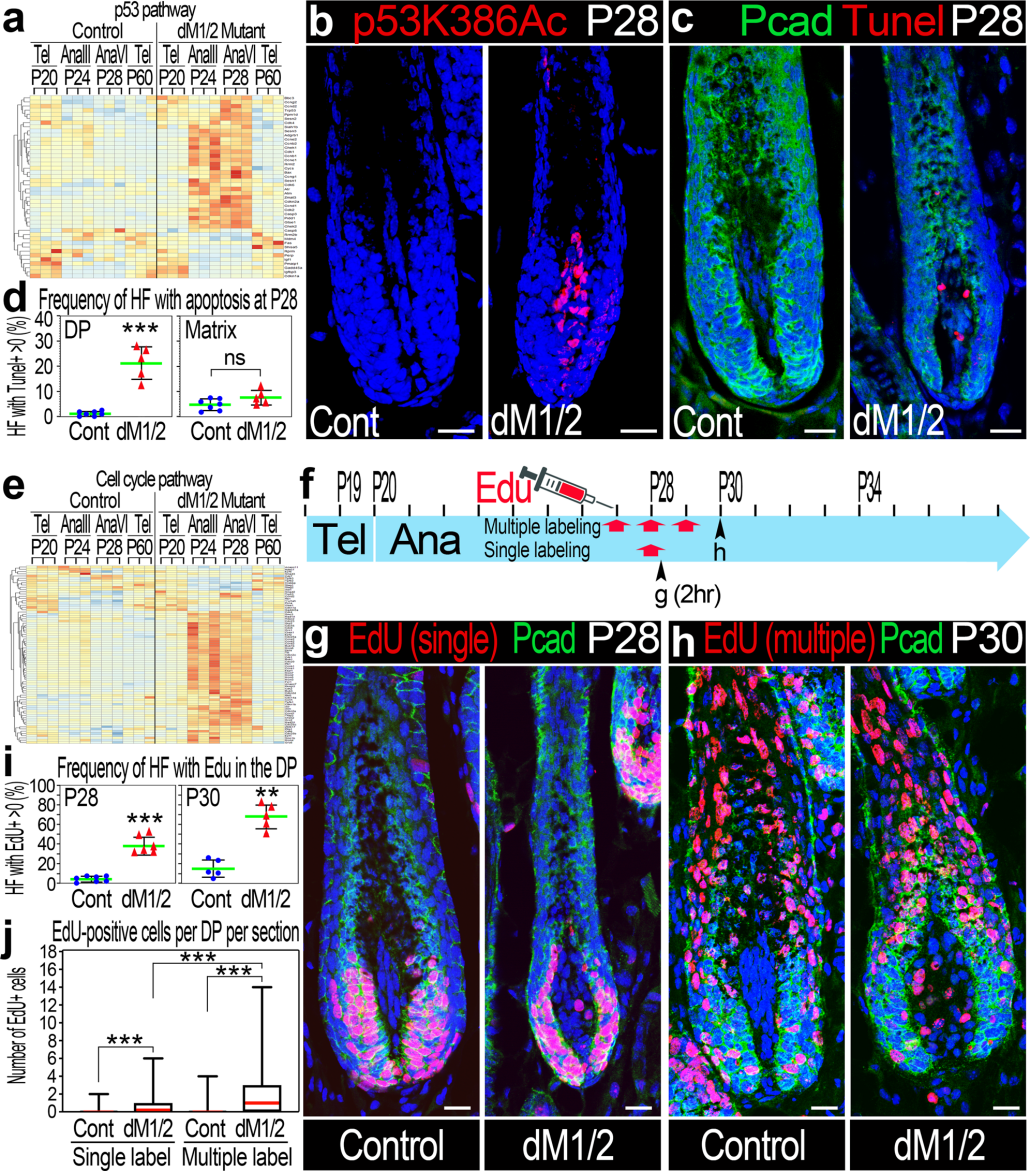
Proliferation and apoptosis concomitantly occur in the DP during anagen in the absence of Hdac1 and Hdac2. **a** Heatmap analysis of altered p53 pathway-associated genes that were chronologically clustered according to the hair cycle. Note that expression of the genes in the pathway is altered in the mutant predominantly during anagen. **b** Immunostaining with an antibody that recognizes specifically the acetylated K386 form of p53 (p53Ac). *n* = 6 mice per genotype. **c** Tunel staining revealed apoptotic cells in the DP of dM1/2 mutant. Co-immunostaining of Pcad was performed to outline the DP. *n* = 5 mice per genotype. **d** Quantification of the number of follicles per mouse (in percentage) with apoptotic cells in the DP (left) and matrix (right). Data are mean ± SD. *n* (Control) = 7 mice, *n* (dM1/2) = 5 mice, and 85-100 follicles per mouse were scored. ***P = 8.28E-06, unpaired two-tailed Student’s *t*-test. **e** Chronological heatmap analysis of altered cell-cycle pathway-associated genes to illustrate the altered expression of these genes in the dM1/2 mutant during anagen. **f** Schematic representation of the EdU labeling experiments. On top, a time scale of postnatal day (P) is shown. Red arrows indicate time of Edu injection. Black arrowheads indicate time of skin harvest and the corresponding image is indicated beneath the arrowhead. **g** EdU incorporation 2 h post injection of a single labeling experiment. Pcad immunostaining was also included to distinguish between DP and matrix cells. DP cells are negative for Pcad. *n* = 5 mice per genotype. **h** EdU incorporation 24 h from the last injection of a multiple labeling experiment. *n* = 5 mice per genotype. **i** Quantification of the number of follicles per mouse (in percentage) with EdU-positive cells in the DP. On the left, the results of the single labeling experiment are presented. *n* (control) = 7 mice, *n* (dM1/2) = 6 mice. On the right, the data of the multiple labeling experiment are summarized. *n* (control) = 5 mice, *n* (dM1/2) = 5 mice. At least 100 follicles per mouse were scored. Data are mean ± SD. ***P = 1.63E-06, **P = 4.9E-05, unpaired two-tailed Student’s *t*-test. **j** Quantification of the number of EdU-positive cells in the DP per follicle. Data are presented by box-and-whisker plots (red midline, median; box, 25th and 75th percentiles; whiskers, minimum and maximum). *n* = 5 mice per genotype and >100 follicles per mouse were scored. ***P < 0.001, unpaired two-tailed Student’s *t*-test. Scale bar in all panels; 20 μm. HF; hair follicle. Source data are provided as a Source Data file.

The biological significance of altered p53 pathway was first investigated. Note that the expression of most of the genes in p53 pathway remain unaltered in controls throughout the hair cycle (Fig. 3a). Elevated expression of these genes is observed only in the mutant and only during anagen. It is important to note that while the RNA-seq analysis probes alterations only at the transcript level, p53 is predominantly under tight regulation post translationally^32^. Since acetylation of p53 is often required to activate p53, and since Hdac1 and Hdac2 were previously shown to regulate p53 acetylation, the acetylation state of p53 in the DP during mid-anagen (P28) was tested by immunostaining with a series of antibodies that specifically recognize different acetylated lysine residues of activated p53 (Supplementary Fig. 5a, b). While acetylated p53 is undetected in control mice, the K386 and K373 acetylated forms of p53 are abundant specifically in mutant DP cells (Fig. 3b; Supplementary Fig. 5c), demonstrating that not only p53 pathway is transcriptionally elevated but also functionally over activated. Furthermore, the acetylation status of p53 during the first cycle was interrogated to corroborate the dispensability of Hdac1/2 during the mid-anagen of the first cycle. Consistently, no acetylated forms of p53 were detected during the first cycle (P12) regardless of the genotype (Supplementary Fig. 5d).

Normally, activated-p53 arrests the cell cycle or promotes apoptosis. To address the outcome of activated-p53 elevation in the DP during the second cycle, Tunel staining was performed at P28 to detect apoptotic cells (Fig. 3c, d). Expectedly, follicles with Tunel+ cells were rarely identified in the matrix of both control and mutant mice (Fig. 3d, right panel). In contrast, while apoptotic cells in the DP are extremely rare events in control mice, follicles with Tunel+ cells in the DP are often detected in mutant mice (Fig. 3d, left panel). This suggests that overactivation of p53 in the DP promotes apoptosis, and Hdac1 and Hdac2 play an important role in protecting DP cells from apoptosis by down modulating p53 activity. To further corroborate the dispensability of Hdac1 and Hdac2 during the first cycle, Tunel staining was also performed at P12. Consistently, Tunel+ cells in the DP could not be detected regardless of the genotype (Supplementary Fig. 6a–c).

### Hdac1 and Hdac2 during anagen maintain the quiescent state of DP cells

The biological significance of alterations in the cell-cycle pathway was next explored. Chronological heatmap analysis of the genes in the pathway (Fig. 3e) illustrated that while similar and low expression levels of these genes persist in control mice throughout the hair cycle, elevated levels of most of these genes in the DP were found in mutant mice only during anagen, suggesting that DP cells proliferate during anagen in the absence of Hdac1 and Hdac2. To test this, Edu labeling experiments during the second cycle were performed (Fig. 3f). A single dose of Edu was ip injected at P28, and 2 h later, the skin was tested for Edu incorporation (Fig. 3f, g). Expectedly, while matrix cells were abundantly labeled, follicles with Edu-labeled DP cells were barely found in control mice (Fig. 3g, i; left panel). However, follicles with Edu-labeled DP cells were readily detected in mutant mice. Remarkably, a fraction of about 40% of the mutant follicles was found Edu-labeled in the DP (Fig. 3i; left panel). Edu experiments were also performed during the first cycle (Supplementary Fig. 7a). Here too, a single dose of Edu was ip administered at P12, and Edu incorporation was tested 2 h post injection. Using Corin as a specific marker for DP, Edu-labeled DP cells were barely detected regardless of the genotype, consistent with the lack of phenotype during the first cycle (Supplementary Fig. 7b–f).

**Fig. 4:**
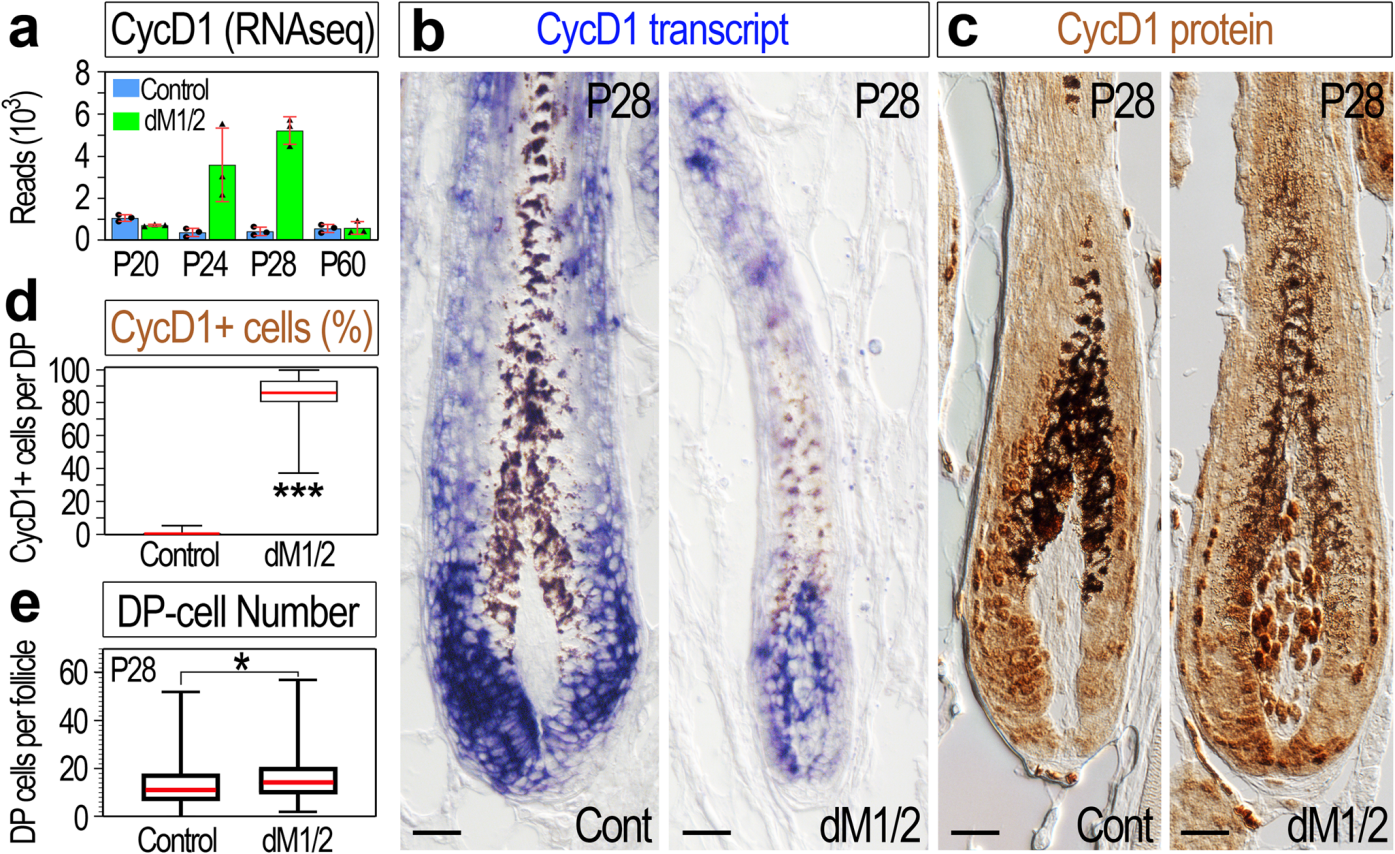
DP-specific ablation of Hdac1 and Hdac2 results in upregulation and nuclear localization of CycD1 in the DP and an increase in DP cell number during anagen. **a** Normalized number of mRNA reads for CycD1 scored by the RNA-seq analysis. Data are mean ± SD. **b** In situ hybridization for CycD1 reveals upregulation of CycD1 transcript (blue) in the DP of dM1/2 mutant. *n* = 3 mice per genotype. Scale bar; 25 μm. **c** Immunohistochemistry of CycD1 unveils nuclear localization of CycD1 specifically in the DP of dM1/2 mutant. Note that a relatively very large mutant follicle is purposely depicted to illustrate the extent of the CycD1-positve cells in the DP. Scale bar; 25 μm. **d** The number of CycD1-positive cells per DP per follicle was scored and presented in percentage. Data are presented by box-and-whisker plots (red midline, median; box, 25th and 75th percentiles; whiskers, minimum and maximum). *n* = 3 mice per genotype and at least 80 follicles per mouse were analyzed. ***P < 0.0001, two-sided Mann–Whitney test. **e** Quantification of the number of DP cells per follicle during anagen. Data are presented by box-and-whisker plots (red midline, median; box, 25th and 75th percentiles; whiskers, minimum and maximum). *n* = 5 mice per genotype and at least 100 follicles per mouse were scored. *P = 0.03, unpaired two-tailed Student’s *t*-test. Source data are provided as a Source Data file.

Of note, despite the dramatic incline in the frequency of mutant follicles with Edu-labeled DP cells during the second cycle, only a few DP cells per follicle were found labeled (Fig. 3g, j; single labeling). To further explore the extent of proliferation in the DP of mutant mice, multiple labeling with Edu was performed (Fig. 3f, h). Edu was ip injected once a day for three days starting at P27, and 24 h after the last injection (P30), Edu incorporation was tested in skin sections (Fig. 3f, h, i; right panel, and j; multiple labeling). While the number of DP-labeled cells per follicle increases, substantial number of DP cells remain unlabeled (Fig. 3h, j). This suggests that either a subpopulation of DP cells proliferate while the rest remain quiescent, or alternatively, the entire population of DP cells is in a proliferative state, but the pace of the cell cycle is extremely slow. To distinguish between these two hypotheses, the cell-cycle gene signature, found to be altered in DP mutant, was revisited. Based on the RNA-seq analysis, the expression of *Ccnd1* (CycD1) during anagen is dramatically elevated in the DP of mutant mice (Fig. 4a). CycD1 is required and plays an important role in the G1 phase of the cell cycle^33^. In situ hybridization and immunostaining for CycD1 revealed that CycD1 in control mice is abundantly expressed in the matrix and not detected in the DP (Fig. 4b, c). In contrast, CycD1 in mutant mice is expressed in both the matrix and DP. Note that close to 90% of DP cells per follicle were nuclear positive for CycD1 in the mutant (Fig. 4d), clearly demonstrating that the proliferative state of the DP is not restricted to a small subpopulation. Together, the Edu labeling experiments and the CycD1 analysis suggest that Hdac1 and Hdac2 play an important role in maintaining the quiescence state of the DP during anagen of the second cycle, and further corroborate the dispensability of Hdac1 and Hdac2 during the first cycle.

### Proliferation in mutant DP is extrinsically induced during anagen while apoptosis in mutant DP is exacerbated during catagen

**Fig. 5:**
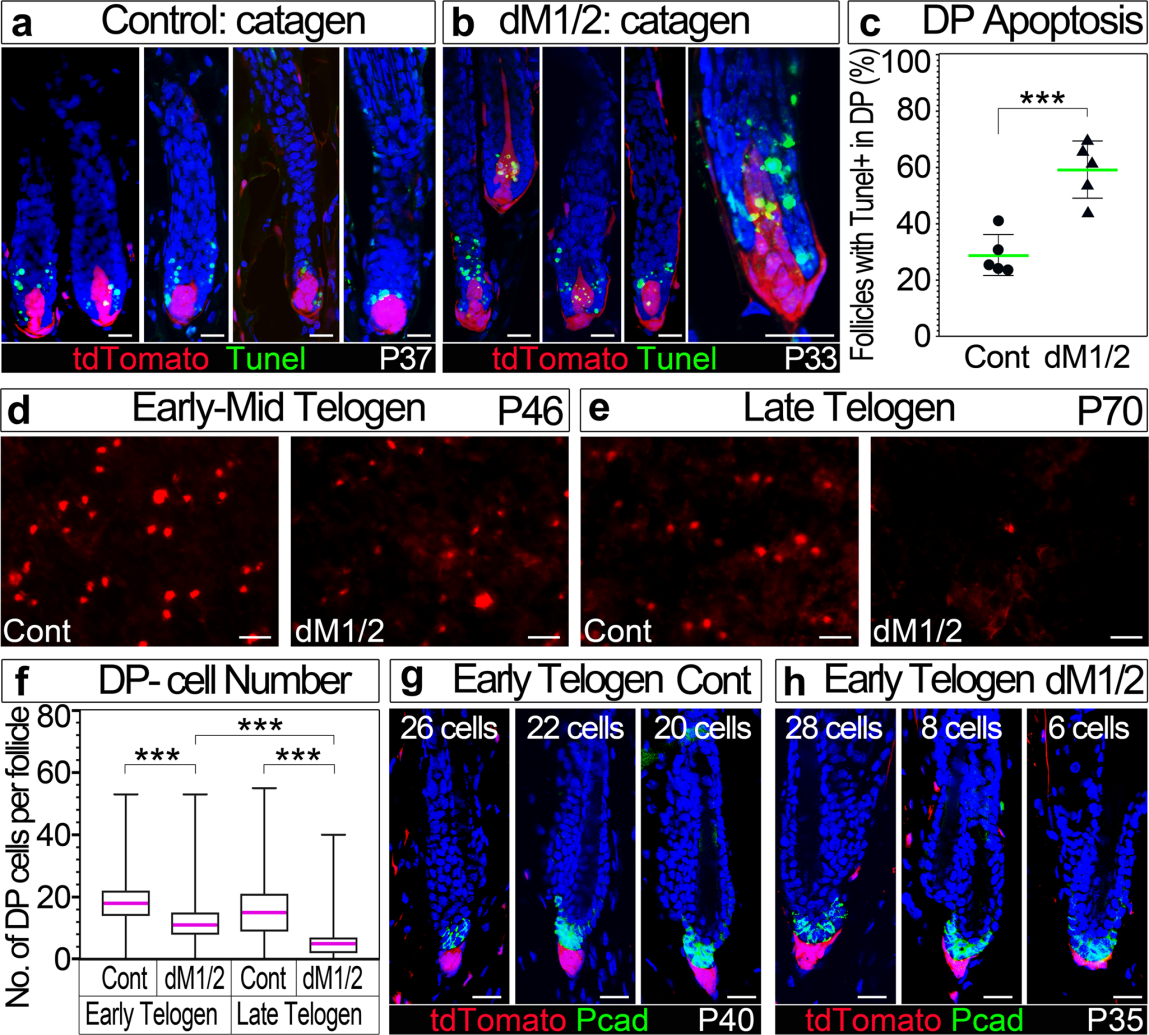
Apoptosis in the DP during catagen is exacerbated in the dM1/2 mutant. **a**, **b** Tunel staining of control (**a**) and dM1/2 mutant (**b**) mice during catagen is shown. The DP is endogenously labeled with tdTomato. Follicles at different stages of catagen are shown for each genotype. In b, the follicle in the right panel was imaged at higher magnification. Scale bar; 20 μm. **c** Quantification of the number of follicles (in percentage) with Tunel-positive cells in the DP. Data are presented by scatter plots with values of individual mice (mean ± SD). *n* = 5 mice per genotype and 50–100 follicles per mouse were scored. ***P = 0.0006, unpaired two-tailed Student’s *t*-test. **d**, **e** Fluorescent imaging of whole skin from the dermis side visualizes the tdTomato-labeled DPs. Note that during early to mid telogen (**d**), the size of the DP in the dM1/2 mutant is smaller, while during late telogen (**e**), most of the DPs in the dM1/2 mutant are too small to be visualized. Scale bar; 100 μm. **f** Quantification of the number of DP cells per follicle during telogen. Note that at early telogen the number of DP cells is reduced from an average of 18 cells per control follicle to 12 cells per dM1/2 mutant follicle. At late telogen, the average number of DP cells per dM1/2 mutant follicle is further reduced to 5. Data are presented by box-and-whisker plots (red midline, median; box, 25th and 75th percentiles; whiskers, minimum and maximum). *n* = 3 mice per genotype and about 100 follicles per mouse were scored. ***P < 0.001, unpaired two-tailed Student’s *t*-test. **g**, **h** Fluorescent images of control (**g**) and dM1/2 mutant (**h**) follicles at early telogen of the second cycle. Pcad immunostaining was performed to label the secondary germ and the ROSA26-tdTomato reporter allele was included to endogenously label the DP. For each genotype, follicles with different number of DP cells (indicated at the upper side of the panel) are shown. *n* = 3 mice per genotype. Scale bar; 20 μm. Source data are provided as a Source Data file.

To address the effect of concomitant proliferation and apoptosis on DP cell number during anagen, DP cell count was performed (Fig. 4e). This analysis illustrates that the number of DP cells increases in mutant mice, suggesting that proliferation is more predominant during anagen. To explore whether the proliferation predominance during anagen is a consequence of the mitogenic environment the DP resides in or alternatively is an intrinsic property of mutant DP cells, Edu labeling and Tunel staining were performed during catagen when the DP is exposed to an apoptotic microenvironment. No Edu labeling was found during catagen in both control and mutant mice, neither in the regressing epithelial strand nor in the DP, suggesting that proliferation in mutant DP is not intrinsic but induced by the microenvironment. Tunel staining however expectedly detected apoptotic cells within the epithelial compartment of the regressing follicles in both control and mutant mice (Fig. 5a, b). Remarkably and in contrast to previous reports^20,21^, apoptosis within the DP of control mice is frequently observed during the catagen phase; about 30% of the follicles exhibit Tunel-positive cells in the DP (Fig. 5c; Supplementary Fig. 8a). Interestingly, the fraction of follicles with Tunel-positive cells in the DP of mutant mice increases dramatically; about 60% of the follicles exhibit apoptosis in the DP (Fig. 5c). Furthermore, the number of DP apoptotic cells per mutant follicle also increases (Supplementary Fig. 8a). This supports the conclusion that during catagen when the surrounding microenvironment is pro-apoptotic, Hdac1 and Hdac2 play a role in reducing the sensitivity of DP cells to the pro-apoptotic cues.

### Hdac1 and Hdac2 maintain the survival of DP cells during the long telogen to allow anagen induction

The lack of proliferation combined with increased apoptosis in mutant DP during catagen predicts reduction in the number of DP cells during regression. Indeed, when the DP is endogenously labeled with tdTomato in both control and mutant mice to allow the detection of a large population of follicles by fluorescent imaging of the DP from the dermis side, follicles with smaller DP were observed during early telogen in mutant mice (Fig. 5d). Remarkably, this reduction in DP size is further exacerbated during this long telogen, and at P70 most of the follicles could not be detected from the dermis side (Fig. 5e). DP cell count in skin sections at both early and late telogen not only corroborated the decline of DP cell number during catagen but also confirmed the persistent loss of DP cells during the long telogen (Fig. 5f–h). This supports that Hdac1 and Hdac2 play an important role in maintaining DP survival during telogen. Of note, the average number of DP cells per mutant follicle at P70 is 5 cells (Fig. 5f), far below the minimal threshold of 10 DP cells required to induce anagen^34^. This suggests that the telogen arrest observed in the mutant at the end of the second cycle is a consequence of the dramatic reduction in DP cell number.

The number of cells in the secondary germ is also important for anagen induction^18^. Using Pcad as a marker for the secondary germ, the number of cells within the secondary germ of mutant mice was compared to controls. No significant difference was observed between control and mutant mice (Supplementary Fig. 8b). Furthermore, immunostaining for structural and stem-cell markers revealed no alterations in stem-cell properties or bulge structure, further corroborating the proposition that reduction in DP cell number in the mutant is the predominant mechanism underlying the telogen arrest.

### P53-dependent and independent apoptosis of DP mutant cells

**Fig. 6:**
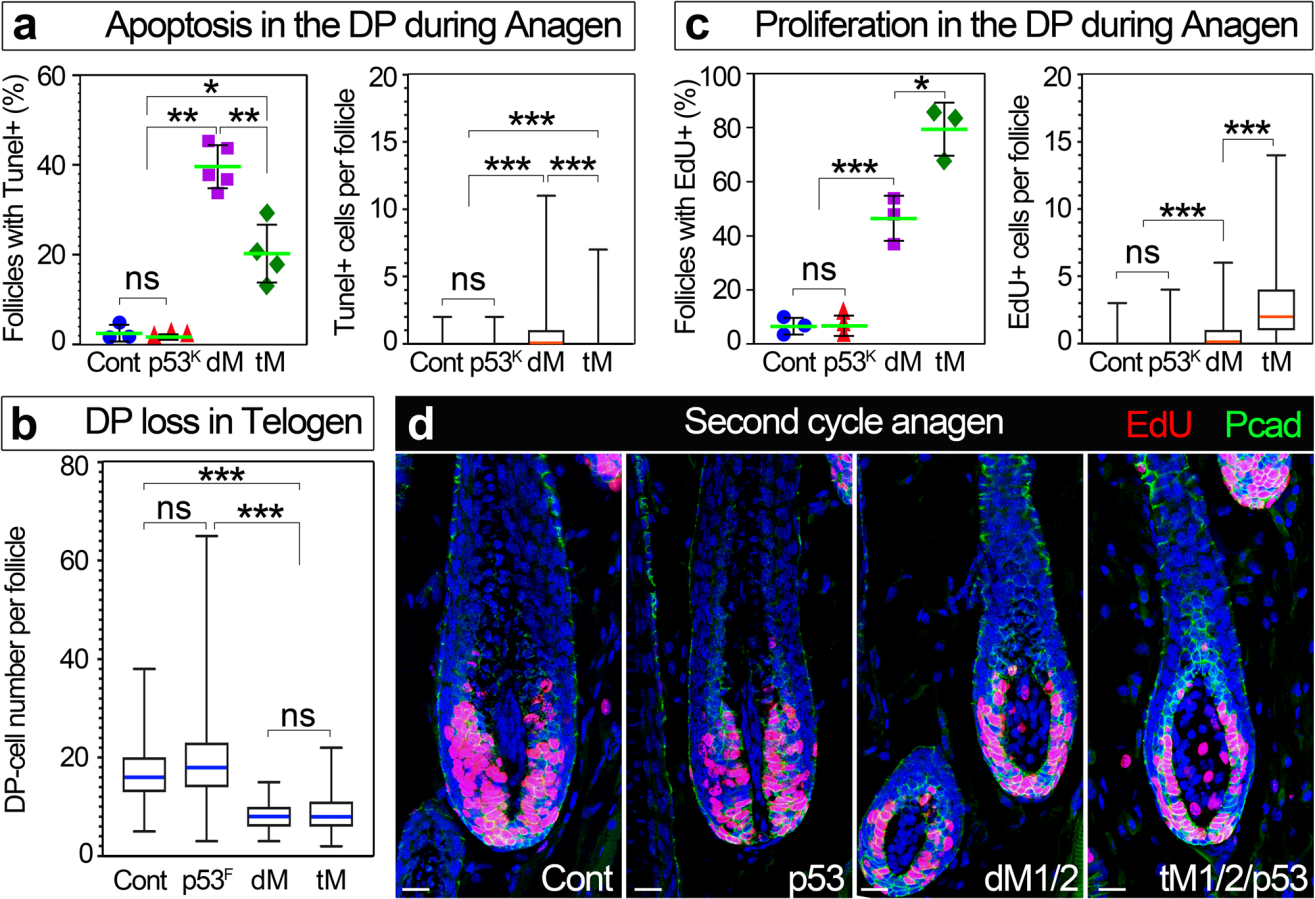
p53 activity in the DP restrains proliferation and promotes apoptosis in a Hdac1/2-dependent mode. **a** Quantification of apoptotic cells in the DP during anagen. On the left, the number of follicles per mouse (in percentage) with Tunel-positive cells in the DP is presented. Data are mean ± SD. ns; not significant, *P = 0.049, **P = 0.004, unpaired two-tailed Student’s *t*-test. On the right, the number of Tunel-positive cells in the DP per follicle is displayed. Data are presented by box-and-whisker plots (red midline, median; box, 25th and 75th percentiles; whiskers, minimum and maximum). ***P < 0.001, unpaired two-tailed Student’s *t*-test. Cont; control (*n* = 3 mice), p53^K^; homozygote for the knockout allele of p53 (*n* = 3 mice), dM; double mutant for Hdac1 and Hdac2 (*n* = 5 mice), tM; triple mutant for Hdac1, Hdac2 and p53 knockout (*n* = 4 mice). 62-106 follicles per mouse were scored. **b** Quantification of the number of DP cells per follicle during telogen (P50). Data are presented by box-and-whisker plots (blue midline, median; box, 25th and 75th percentiles; whiskers, minimum and maximum). ns; not significant, ***P < 0.001, unpaired two-tailed Student’s *t*-test. Cont; control (*n* = 3 mice), p53^F^; homozygote for the conditional (floxed) allele of p53 (*n* = 3 mice), dM; double mutant for Hdac1 and Hdac2 (*n* = 3 mice), tM; triple mutant for Hdac1, Hdac2 and p53 conditional knockout (*n* = 5 mice). About 100 follicles per mouse were scored. **c** Quantification of proliferating cells in the DP during anagen by EdU incorporation. On the left, the number of follicles per mouse (in percentage) with EdU-positive cells in the DP is shown as a mean ± SD. ns; not significant, *P = 0.011, ***P = 0.001, unpaired two-tailed Student’s *t*-test. On the right, the number of EdU-positive cells in the DP per follicle is displayed as a box-and-whisker plot (red midline, median; box, 25th and 75th percentiles; whiskers, minimum and maximum). ***P < 0.0001, unpaired two-tailed Student’s *t*-test. *n* = 3 mice per genotype and 63–103 follicles per mouse were scored. **d** EdU incorporation during the anagen phase of the second cycle 2 h post injection. Pcad immunostaining was included to distinguish between DP and matrix cells. DP cells are negative for Pcad. Scale bar; 20 μm. Source data are provided as a Source Data file.

While p53 is known to play a role in promoting apoptosis, programed cell death is also induced by other mechanisms. To explore the role of p53 overactivation in apoptosis of DP mutant cells, a triple mutant of *p53*, *Hdac1* and *Hdac2* was generated (designated tM1/2/p53). Initially, a knockout p53 mouse line was used to generate the triple mutant in order to exclude the dilution effects that multiple floxed alleles may have on cre activity and consequently reduce the deletion efficiency. Crosses were designed to obtain 4 distinct genotypes; controls, single *p53*, double *Hdac1/2* and triple *Hdac1/2/p53* mutants. Tunel staining during mid-anagen of the second cycle revealed that while apoptosis in the DP of the triple mutant is reduced compared to the dM1/2 double mutant (Fig. 6a), some DP cells still apoptose. This suggests that elevation and overactivation of p53 in the double mutant contributes to the apoptosis within the DP, but p53-independent alterations are also involved in promoting apoptosis. Note that p53 knockout mice are indistinguishable from controls, further corroborating that overactivation of p53 during anagen contributes to apoptosis in the double mutant. Similar experiments were also performed with a floxed allele of *p53* to generate a triple mutant in which *p53* was ablated specifically in the DP (Supplementary Fig. 9a). Similar reduction of apoptosis in the triple mutant was observed (Supplementary Fig. 9a), suggesting that the reduction in apoptosis is DP specific and not a consequence of alterations in the epithelial compartment that may occur as a result of germ line ablation of *p53*.

The reduction in DP cell number during the telogen of the second cycle in the dM1/2 double mutant (Fig. 5f) is inconsistent with the RNA-seq analysis, which clearly demonstrates that elevation of p53 pathway in the dM1/2 mutant occurs predominantly during anagen and not telogen (Fig. 3a), suggesting that the reduction in DP cell number during telogen is p53 independent. Indeed, reduction in DP cell number during telogen occurs in the triple mutant at the same magnitude observed in the double mutant (Fig. 6b), confirming that the role of Hdac1 and Hdac2 in retaining the number of DP cells during telogen is not mediated through the regulation of p53.

### Hdac1 and Hdac2 in the DP cell-autonomously suppress proliferation to maintain the quiescent state of the DP

The role of Hdac1 and Hdac2 in protecting cells from apoptosis through the regulation of p53 was previously reported and well documented in other settings^35–37^. In addition, Hdac1 and Hdac2 were previously shown to promote proliferation^38^. Our finding that Hdac1 and Hdac2 in the DP also play an important role in suppressing proliferation and thus maintaining niche quiescence has never been described previously. While this finding suggests a cell-autonomous role of Hdac1 and Hdac2 in suppressing the cell cycle in the DP, a compensatory response within the DP that induces proliferation as a consequence of nearby dying cells is also a possibility. The compensation hypothesis predicts that proliferation in mutant DP is proportional to apoptosis, and therefore, reduction in apoptosis observed in the triple mutant should result in proportional reduction of proliferation. However, Edu labeling during mid-anagen unveiled that proliferation within the DP not only persists but also increases in the triple mutant despite the reduction in apoptosis (Fig. 6c, d). This increase in proliferation occurs whether a knockout or a conditional allele of *p53* was used and ultimately leads to increase in DP size (Supplementary Fig. 9b, c). Together these data refute the compensation hypothesis and suggest that (1) Hdac1 and Hdac2 in the DP play an important role in regulating cell-autonomously the proliferative state of the DP; and (2) elevation and overactivation of p53 in the double mutant restrains proliferation presumably through its function in cell-cycle arrest.

### Hdac1 and Hdac2 play a role in maintaining Wnt activity in the DP during anagen

**Fig. 7:**
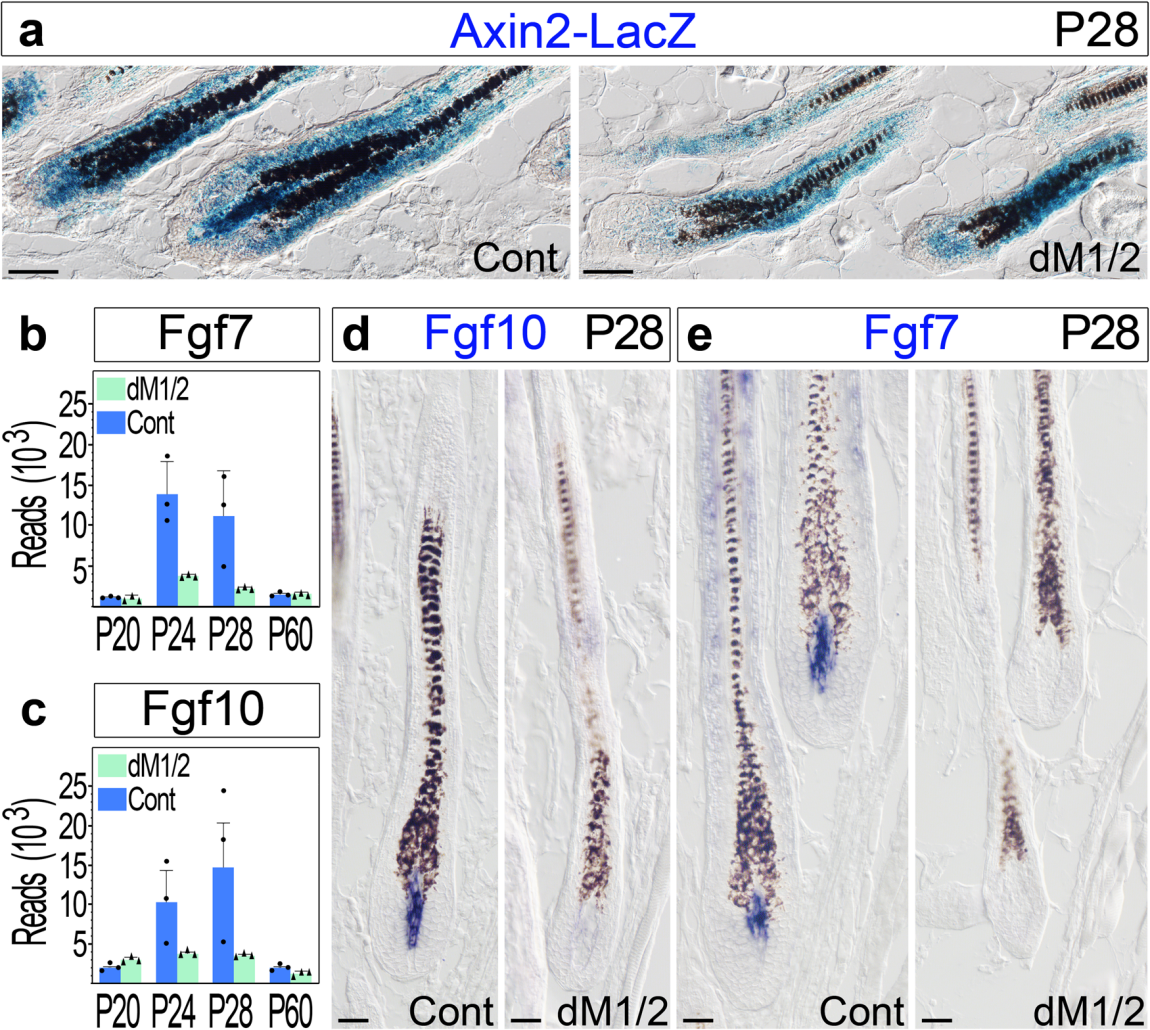
Abrogation of Hdac1 and Hdac2 in the DP results in reduced Wnt signaling activity during anagen in both the DP and matrix. **a** X-gal staining of skin sections from control and dM1/2 mutant mice at P28 shows LacZ expression. *n* = 6 mice per genotype. Scale bar; 50 μm. **b**, **c** Normalized number of mRNA reads for Fgf7 (**b**) and Fgf10 (**c**) derived from the RNA-seq analysis presented in Fig. 2. Both Fgf7 and Fgf10 are known target genes of Wnt signaling activity in the DP. Data are mean ± SD, *n* = 3 biologically independent replicates. **d**, **e** In situ hybridization for Fgf10 (**d**) and Fgf7 (**e**) corroborated the reduction in the expression of these genes in the DP of the dM1/2 mutant. *n* > 3 mice per genotype. Scale bar; 25 μm. Source data are provided as a Source Data file.

It is important to note that hair thinning, observed in both the double and triple mutants at the end of the second cycle (Fig. 1b; Supplementary Fig. 1f), is inconsistent with the known direct correlation between DP size and hair dimensions^34,39,40^. Remarkably, hairs thin in the mutants despite the increase in DP cell number during anagen. Furthermore, in the absence of Hdac1 and Hdac2 in the DP, anagen shortens (Fig. 1k). These suggest that in addition to the maintenance of DP size, Hdac1 and Hdac2 play important role in regulating signaling pathways in the DP that control hair dimensions and anagen duration. Indeed, these phenotypic outcomes resemble those observed when b-catenin was ablated specifically in the DP^26,31^. To explore whether Hdac1 and Hdac2 play a role in regulating Wnt activity in the DP, the *Axin2-lacZ* Wnt-reporter allele was introduced in both dM1/2 mutant and control mice. X-gal staining of P28 skin sections from dM1/2 mutant and control mice revealed that Wnt signaling activity is reduced in both the DP and matrix of mutant mice (Fig. 7a). Furthermore, expression of both *Fgf7* and *Fgf10* is reduced in the DP of dM1/2 mutant during mid-anagen (Fig. 7b–e), consistent with a previous report illustrating that both *Fgf7* and *Fgf10* are target genes of Wnt signaling in the DP^26^. We have recently demonstrated that the Wnt agonists *Rspondins* are predominantly expressed in the DP, are target genes of Wnt signaling in the DP, play an important role in augmenting Wnt activity in the matrix, and consequently, regulate the duration of anagen^31^. Revisiting the RNA-seq data unearthed that expression of all 4 *Rspondins* in the DP of dM1/2 mutant during mid-anagen is reduced (Supplementary Fig. 10a). This was further corroborated by in situ hybridization (Supplementary Fig. 10b). Together these data suggest that Hdac1 and Hdac2 in the DP are required during anagen to maintain Wnt activity in the DP, and consequently, regulate the duration of anagen and hair shaft dimensions.

## Discussion

The “stem-cell niche” hypothesis, proposed four decades ago by Schofield^41^, describes the physiologically limited microenvironment that nurtures and regulates stem-cell activity and number, and generally refers to the stem-cell location. Since then, the effects of the instructive and inductive signals provided by the niche on stem-cell behavior were extensively studied^1^. However, the effects of the same signals on the cells that constitute the niche were barely explored, and largely assumed negligible because of the differentiation state of these cells. Furthermore, accumulating evidence clearly demonstrate that the niche is structurally complex and composed of multiple components and cell types^42,43^. While this complexity must be intricately maintained to guard stem-cell function, this maintenance sometimes requires morphological adaptations to periodically restructure the morphology and position of the niche. Such is the case with the mesenchymal niche of the hair follicle, the DP. By ablating both *Hdac1* and *Hdac2* specifically in the DP, we unveil the important role these regulators play in the maintenance of DP functionality and integrity during the hair cycle and provide new insights onto the mechanism by which the DP remains responsive to the signals that regulate progenitor and stem-cell activity but repurposes the transduction of these signals towards different readouts and functions.

While the DP plays important and diverse roles in regulating different aspects of follicle biology during the hair cycle, the mechanisms that maintain the functionality of DP cells despite the extreme alterations in the microenvironment these cells are exposed to due to the cyclic nature of the hair follicle remain poorly understood. During anagen, the proliferation rate of matrix cells is extremely high (see for example Fig. 3g). Such proliferative microenvironment requires the presence of high levels of mitogenic signals. While this overload of mitogenic cues is essential for the production and growth of the hair shaft, it poses a difficult challenge to non-dividing cells such as the DP. Furthermore, such a microenvironment results in mitogenic stress that often induces protective mechanisms to restrict proliferation^44^. One known mechanism is activation of p53 that promotes cell-cycle arrest or apoptosis^45^. While such protective mechanisms may be beneficial and required to balance and fine tune the proliferative capacity of matrix cells, it may lead non-dividing DP cells to undergo apoptosis and consequently abrogate DP functionality. Such challenges the DP faces are further complicated by our recent findings that mitogenic signals such as Fgf and Wnt ligands are required to regulate the hair-cycle clock in the DP^31^, rendering the capacity of DP cells to express the appropriate receptors to sense the mitogenic signals an essential part of DP functionality.

Here we provide new evidence to support a mechanism employed by the DP to repurpose the conventional role of mitogenic signaling in promoting proliferation towards regulating a different biological function while simultaneously maintaining DP quiescence and survival. Concomitant ablation of *Hdac1* and *Hdac2* specifically in the DP during anagen results in proliferation and apoptosis of DP cells, illustrating that quiescence and survival of DP cells are maintained by a cell-autonomous mechanism that does not intervene with the ability of DP cells to sense the microenvironment. This allows DP cells to respond to the mitogenic signals but also to uncouple the standard role of these transducing signals in proliferation from their roles in other biological processes. Quiescent cells require the induction of type D cyclins (CycD) to enter the cell cycle^33^. Remarkably, CycD1 is induced and translocated to the nucleus of DP cells lacking Hdac1 and Hdac2, suggesting that Hdac1 and Hdac2 in the DP suppress the expression of CycD1 and consequently prevent DP cells from entering the cell cycle.

Concomitant to proliferation of DP cells, apoptosis in the DP during anagen also occurs when *Hdac1* and *Hdac2* are abrogated specifically in the DP. While apoptosis in DP mutant may be induced as a response to pro-apoptotic signals produced by nearby proliferating cells to balance cell number and thus does not reflect an intrinsic role of Hdac1/2 in protecting the DP from apoptosis, the complete lack of proliferation and the exacerbated apoptosis in the mutant DP during catagen argues against this cell non-autonomous mechanism. Of note, while mitogenic signaling through activation of the Erk1/2 MAPK pathway often leads to proliferation, activation of the Erk1/2 MAPK pathway under certain circumstances may also result in apoptosis^44,46^. Activation of Erk1/2 MAPK pathway in normal primary cells by oncogenic Ras for example elicits cell-cycle arrest and apoptosis^47^. This activation results in transcriptional upregulation of Dmp1 that binds and activates the promoter of Arf^48^, which leads to cell-cycle arrest and apoptosis by p53-dependent and independent modes of action^49^. Interestingly, both p53-dependent and independent apoptosis in DP lacking Hdac1 and Hdac2 occurs during anagen, and Arf is also elevated in the DP of double Hdac1/2 mutant. Together, these data suggest that Hdac1 and Hdac2 protect DP cells from undergoing apoptosis in response to the impinging mitogenic stress by directly deacetylating p53 and reducing its activity and by indirectly inhibiting p53-independent processes that promote apoptosis.

In contrast to anagen, the DP is directly exposed to a pro-apoptotic microenvironment during the catagen phase but yet remains largely resistant to apoptosis in wild-type mice^20^. In the absence of Hdac1 and Hdac2 in the DP, apoptosis within the DP compartment during the catagen phase dramatically increases, leading to a substantial reduction in the number of DP cells when the telogen phase ensues. Remarkably, DP cell loss continues during the long telogen even though the microenvironment of the quiescent follicle during telogen does not normally promote apoptosis. Furthermore, this DP cell loss still occurs even when p53 is specifically ablated in the DP in addition to Hdac1/2 abrogation, suggesting that Hdac1 and Hdac2 in the DP support DP survival during the telogen phase in a p53-independent manner.

The number of DP cells is tightly regulated and maintained. Despite the fluctuation in the number of DP cells between anagen and telogen, very little proliferation or apoptosis are normally observed within the DP, suggesting that alterations in DP cell number during the hair cycle are the consequence of the cyclic mobilization of DP cells between the DP compartment and the dermal sheath^20,21^. Our findings suggest that the number of DP cells is maintained by the regulation of Hdac1 and Hdac2 in the DP. Furthermore, by expressing the diphtheria toxin in the DP to selectively ablate DP cells, the number of DP cells was previously reduced in-vivo to evaluate its effect on hair shaft formation and cycle^34^. When the number of DP cells was moderately reduced, hair length and thickness were decreased, demonstrating that the number of DP cells dictates hair size and structure. In contrast, when the DP cell number was dramatically reduced beneath 10 cells per follicle, follicles were arrested in telogen^34^. Remarkably, the predominant proliferation within the DP during the anagen phase in the absence of Hdac1 and Hdac2 results in increase of DP cell number, but hair length and thickness were reduced, emphasizing the importance of the balance between DP cell number and proper function of DP cells. However, the dramatic reduction in DP cell number during telogen when *Hdac1* and *Hdac2* were ablated in the DP results in telogen arrest, consistent with the critical threshold of DP cell number required to induce anagen.

In the absence of Hdac1 and Hdac2 in the DP, Wnt signaling activity in both the DP and matrix is reduced. We have recently shown that Wnt signaling activity in the matrix depends on Wnt activity in the DP^31^. This dependency allows both compartments to maintain the anagen phase, and the DP to regulate the duration of anagen. Mechanistically, this dependency relies on a positive feedback loop in which Wnt signaling in the DP activates the expression of *Rspondins* that augment Wnt signaling in the matrix^31^. Indeed, the expression of all 4 *Rspondins* was diminished in the DP of the dM1/2 mutant, corroborating the reduction of Wnt signaling activity in the DP and suggesting that Hdac1 and Hdac2 sustain high levels of Wnt activity in the DP and consequently orchestrate Wnt activity in the matrix. Furthermore, Wnt activity in the DP was previously shown to control the expression of *Fgf7* and *Fgf10* ^26^, which are thought to govern Fgf signaling in the matrix and control hair dimensions. Consistently, both *Fgf7* and *Fgf10* are reduced in the DP of dM1/2 mutant. While the mechanism by which Hdac1/2 regulate Wnt activity levels in the DP remains to be elucidated in future studies, the data provided in the current study illustrate that the capacity of Hdac1 and Hdac2 to monitor the positive feedback loop of Wnt signaling not only coordinates the duration of anagen but may also provide molecular means to supervise the dimensions of the hair shaft.

The dispensability of Hdac1 and Hdac2 in the DP during the first cycle is puzzling once the role of Hdac1 and Hdac2 in protecting DP cells from proliferation signals during anagen and pro-apoptotic cues during catagen is considered. Furthermore, the essential role of Hdac1 and Hdac2 in maintaining Wnt signaling activity in the DP and the important role of Wnt signaling in maintaining the anagen phase are further inconsistent with the lack of hair phenotype during the first cycle despite efficient deletion of *Hdac1* and *Hdac2*. While the expression of other Hdacs was not tested in the current study, it is likely that functional redundancy between Hdac1 and Hdac2 to other Hdacs that are predominantly expressed in the DP during the first but not subsequent cycles allow normal anagen of the first cycle. Of note, this redundancy is likely to be restricted to only a subgroup of genes that mediate the function of Hdac1 and Hdac2 in the DP, as increased chromatin acetylation in the DP lacking Hdac1 and Hdac2 is observed already during late anagen of the first cycle. Furthermore, since the cre of the Corin-cre line used in the current study is active postnatally, the current study does not exclude the possibility that Hdac1/2 in the DP may play a role in hair-follicle morphogenesis during embryogenesis.

While redundancy may contribute to the lack of phenotype during the first cycle, epigenetic memory restricted to a subgroup of genes and to only a limited period of the anagen phase is also consistent with a normal first cycle despite the absence of Hdac1 and Hdac2 in the DP. We propose that the protective roles of Hdac1 and Hdac2 are acquired during very early anagen, and once these roles are set in action, Hdac1 and Hdac2 are no longer required. For example, the expression of cell-cycle genes in the DP may be repressed during early anagen by the chromatin deacetylation activity of Hdac1 and Hdac2, and this repression is subsequently maintained by other mechanism during mid and late anagen. Such maintenance might be achieved epigenetically by histone methylation of the deacetylated lysine groups and lasts only for the anagen phase. During telogen, this epigenetic maintenance is erased, and during regeneration, Hdac1 and Hdac2 are again required to epigenetically repress the expression of cell-cycle genes. This reversible two-step mechanism of epigenetic acquisition and maintenance is consistent with the lack of hair phenotype and the timely restricted cre activity of our mouse model during the first cycle. During the early anagen of the first cycle, the cre activity of the Cor-cre allele is negligible and reaches its maximal activity only during mid-anagen (P8) of the first cycle^26^. While *Hdac1* and *Hdac2* are efficiently abrogated during mid to late anagen of the first cycle, their role has been already established and maintained, and they are no longer required at this stage to protect the DP. However, as the repressive activity of Hdac1 and Hdac2 at the chromatin level is somehow erased during telogen, the absence of Hdac1 and Hdac2 in the DP during the early anagen of the next cycle results in malfunction of DP cells and consequently hair phenotype. Future analysis is required to test this hypothesis.

## Methods

### Mice

The CMV-cre transgenic mouse line^50^ (Stock No. #006054, C57BL/6J), the p53 conditional knockout line^51^ (Stock No. #008462, C57BL/6J), the reporter mouse lines ROSA26 tdTomato^52^ (Stock No. #007909, C57BL/6J) and ROSA26 EYFP^53^ (Stock No. #006148, C57BL/6J), and the Axin2-lacZ Wnt-reporter line^54^ (Stock No. #005304, C57BL/6J) were obtained from Jackson Laboratory. Hdac1 and Hdac2 conditional knockout mice^27^ (C57BL/6J) were kindly provided by Eric Olson (The University of Texas Southwestern Medical Center). The DP-specific Corin-cre mouse line (FVB) was previously generated^26^ and kindly provided by Bruce Morgan (Harvard medical school). To establish the p53 knockout line, the p53-floxed mouse line was crossed with the CMV-cre line that ubiquitously expresses the cre recombinase in all cells. Progeny were out-crossed to exclude the CMV-cre allele. Mice were housed according to the Federation of Laboratory Animal Science Associations (FELASA) guidelines. Mice were bred and maintained in a temperature-controlled room, on a 12 h light–dark cycle, with food and water available ad libitum. All animal protocols were approved by the Institutional Animal Care and Use Committee (IACUC) at Bar-Ilan University.

Telogen of the first hair cycle in males lasts for only two days while in females continues longer and varies. When the telogen of the first cycle was analyzed, dorsal skins from P20 females were collected to avoid stage unambiguity. When the anagen of the second cycle was analyzed, only males were used to exploit the synchronized nature of their anagen induction and thus minimize hair-cycle variation. The telogen of the second cycle lasts for weeks, and thus both males and females were analyzed.

Crosses for the Hdac1/2 analysis were designed to obtain the following genotypes:

Controls: [Cor-cre/+; Hdac1Flox/+; Hdac2Flox/+] OR [Cor-cre/+; Hdac1Flox/+; Hdac2+/+] OR [Cor-cre/+; Hdac1+/+; Hdac2Flox/+] OR [Cor-cre /+; Hdac1Flox/+; Hdac2Flox/Flox] OR [Cor-cre/+; Hdac1Flox/Flox; Hdac2Flox/+]

Double mutants (dM1/2): [Cor-cre/+; Hdac1Flox/Flox; Hdac2Flox/Flox]

Crosses for the triple mutant (tM1/2/p53) analysis were designed to obtain the following genotypes:

Controls: [Cor-cre /+; Hdac1Flox/+; Hdac2Flox/Flox; p53Flox/+] OR [Cor-cre /+; Hdac1Flox/Flox; Hdac2Flox/+; p53Flox/+]

Double mutants (dM1/2): [Cor-cre/+; Hdac1Flox/Flox; Hdac2Flox/Flox; p53Flox/+]

Triple mutants (dM1/2/p53): [Cor-cre/+; Hdac1Flox/Flox; Hdac2Flox/Flox; p53Flox/Flox]

### Hair-type analysis

Hairs were plucked at the end of the first and second cycles (P20 and P50, respectively). For hair type analysis, hair shafts were placed on a two-sided adhesive tape slides and were counted under the Zeiss stereomicroscope (Discovery.V12). The analysis was performed on both mutant and control mice (*n* = 5 each), and at least 200 hairs were scored per mouse. To photograph hair shafts, plucked hairs were randomly mounted on slides in a thin layer of Gelvatol and were imaged in bright field with Zeiss upright AxioImagerM2 through a 20X objective. For hair length analysis, hair shafts were placed on a two-sided adhesive tape slides and imaged with Zeiss slide scannerZ1 through a 20x objective. The length was measured with Carl Zeiss’ image analysis tool for Zen Blue edition. 5 mice of each genotype were used, and ten hairs for each hair type were measured per mouse.

### Immunofluorescence, immunohistochemistry, and histology

Depending on the primary antibody, fixed or fresh frozen methodologies were used. To prepare fixed frozen samples, dorsal skins were harvested, fixed in 4% paraformaldehyde (PFA) in PBS for overnight at 4 °C, dehydrated in increasing sucrose concentrations (10%–15%–20%), embedded in Optimal Cutting Temperature (OCT) compound, frozen on liquid nitrogen, and cryosectioned (8–10 μm) with Cryostat Leica CM3050S. To prepare fresh frozen samples, dorsal skins were first stretched on a PVDF membrane to keep it flat and then frozen in OCT.

When no antigen retrieval was required, sections were fixed for 10 min in 4% PFA, washed twice for 10 min each (2 × 10 min) in PBS, permeabilized for 10 min in PBS+0.05% Tween (PBST), and blocked for 2 h in 10% heat inactivated sheep serum (HISS) in PBS. Sections were incubated overnight at 4 °C with the primary antibody, washed (3 × 10min) in PBS, and incubated for 1 h at room temperature with the secondary antibody. Sections were then washed (3 × 10min) in PBS and mounted with DAPI FluoroMount-G (Electron microscopy sciences cat#17984-24). When antigen retrieval is required, sections were first fixed for 5 min on ice-cold acetone/methanol (1:1), and then boiled in citrate buffer (pH 6) in a microwave for 5 min for antigen retrieval, cooled to room temperature, permeabilized for 10 min in methanol, washed (3 × 10min) in PBS, and blocked for 2 h in 10% HISS in PBS. Primary and secondary antibodies were used as described for the protocol without antigen retrieval. When using mouse primary antibody, an additional blocking step was performed before incubation with the secondary antibody.

Immunohistochemistry was conducted using the ImmPRESS® HRP Goat Anti-Rabbit IgG (Peroxidase) Polymer Detection Kit (Vector Laboratories cat#MP-7451) according to the manufacturer’s instructions. Sections were fixed, antigen retrieved, washed (3 × 10min) in PBS, and blocked in 2.5% normal goat serum for 2 h. Sections were incubated overnight at 4 °C with the primary antibody, washed (3 × 10 min) in PBS, incubated for 30 min at room temperature with ImmPRESS Polymer Reagent (containing the secondary antibody), washed (3 × 10 min) in PBS and incubated for 2.5 h with Peroxidase (HRP) Substrate solution (ImmPACT DAB, Vector Laboratories cat#SK-4105) to yield an insoluble brown-colored product.

To analyze morphology, fixed sections were stained with Hematoxylin and Eosin (HE) using standard methods (Thermo scientific, Richard Allan scientific cat#7231 and cat#7111, respectively).

*Primary antibodies (star indicates that antigen retrieval is required)*: Guinea pig polyclonal anti-K14 (1:500, Acris #BP-5009), Rabbit polyclonal anti-HDAC1 (1:200*, Abcam #ab19845), Rabbit monoclonal anti-HDAC2 (1:500, Abcam #ab32117), Rabbit polyclonal anti-H3K9Ac (1:1000, Abcam #ab10812), Rabbit polyclonal anti-p53K386Ac (1:200, Abcam # ab52172), Rabbit monoclonal anti-p53K370Ac (1:100, Abcam #ab183544), Rabbit monoclonal anti-p53K373Ac (1:3200*, Abcam #ab62376), Rabbit polyclonal anti-p53K381Ac (1:100, Abcam #ab61241), Rabbit monoclonal anti-p53K382Ac (1:100, Abcam #ab75754), Rabbit polyclonal anti-Corin (1:800, Enshell-Seijffers et al. 2010), Rabbit monoclonal anti-Cycin D1 (1:100*, Cell signaling #2978S), Mouse monoclonal anti-hair cortex Cytokeratin (clone AE13) (1:100*, Abcam #ab16113), Rabbit polyclonal anti-Trichohyalin (1:100*, Santa cruz # sc-98968), Mouse monoclonal anti-GATA3 (clone HG3-31) (1:200*, Santa cruz #sc-268), Rat monoclonal FITC-conjugated anti-CD34 (clone RAM34) (1:50*, eBioscience #11-0341), Rabbit polyclonal anti-SOX9 (1:250*, Millipore #AB5535), Rat monoclonal anti-P-Cadherin (Pcad) (clone 106020) (1:100*, BD #MAB761), Rabbit polyclonal anti-K6 (1:500*, Covance #PRB-169P), Mouse monoclonal anti-Nfatc1 (clone 7A6) (1:50*, Santa cruz #sc-7294, 7A6), Mouse monoclonal anti-K15 (clone LHK15) (1:100*, Thermo scientific #MS-1068), Rabbit polyclonal anti-Phospho-Histone 3 (1:250, Abcam #ab 5176).

*Secondary antibodies*: Donkey anti Guinea pig-Cy5, (1:500, Jackson immunoresearch #706-175-148), Donkey anti-Rabbit-TRITC, (1:1000, Jackson immunoresearch #711-025-152), Donkey anti-Rabbit-FITC, (1:1000, Jackson immunoresearch, #711-095-152), Donkey anti mouse-Alexa Fluor 488, (1:1000, Jackson immunoresearch, #715-545-150), Donkey anti Rat-Cy5, (1:1000, Jackson immunoresearch, #712-175-753)

### Quantification of DP cells

To quantify DP cells, three approaches were used to distinguish between the DP and the epithelial part of the hair follicle depending on the specific stage of the hair cycle, the genotype, and the specific feature scored. To distinguish between DP cells and keratinocytes throughout the hair cycle, DP cells were endogenously labeled with YFP or tdTomato whenever ROSA reporter alleles are genotypically present and the particular protocol used does not affect the integrity of the fluorescent proteins such as antigen retrieval and protease K treatment. When the use of ROSA reporters is not available, Corin or Pcad immunostaining were employed. During anagen, either immunostaining for Corin that specifically marks anagen DP cells or immunostianing for Pcad that specifically labels matrix cells were used. During catagen and telogen, Pcad immunostaining was used to specifically label the epithelial strand or the secondary germ, respectively. Since the dM1/2 mice have a shorter anagen phase, age-unmatched skin sections with stage-matched follicles were used to score DP cells during catagen (control P37, dM1/2 P33) and early telogen (control P40, dM1/2 P35).

### In situ hybridization

Non-radioactive in situ hybridization of fixed sections from dorsal skins was performed with Dig-labeled RNA probes corresponding to nucleotides (nts) 592-931 of Ccnd1 (GenBank Acc. No. NM_007631), nts 465-943 of Fgf7 (NM_008008), nts 699-1211 of Fgf10 (NM_008002), nts 717-1213 of Rspo1 (NM_13868), nts 754-1169 of Rspo2 (NM_001357956), nts 921-1348 of Rspo3 (NM_028351), and nts 300-760 of Rspo4 (NM_001040689).

To generate the probes, plasmids containing the corresponding templates were linearized and used for in vitro transcription with T7 RNA polymerase (Roche cat#10881767001) in the presence of Dig-labeled UTP (DIG RNA Labeling Mix, Roche cat#11277073910). BMpurple substrate (Roche, cat#11442074001) was used for signal detection and Immu-Mount mounting medium (Thermo Fisher Scientific cat#9990402) was used for mounting.

### 5-ethynyl-2’-deoxyuridine (EdU) labeling

To detect proliferation, mice were intraperitoneally (i.p.) injected with 100 μg /g of EdU (Invitrogen cat#C10269) and sacrificed 2 h post injection. To test the extent of proliferation in the DP, mice were i.p injected with 100 μg /g EDU once a day for 3 successive days and then sacrificed 24 h post last injection. To assess the proliferation rate of matrix cells, mice were i.p injected with various EdU concentrations (100 μg/g, 50 μg/g, 25 μg/g, 12.5 μg/g, 7 μg/g, 3 μg/g, 1.5 μg/g) and harvested 30 min or 15 min post injection. This methodology is designed to detect small differences in proliferation rate of matrix cells. EdU detection was performed using the Click-iT reaction Alexa Fluor 555 or 647 Kit (Invitrogen #A10044) according to the manufacturer’s instructions.

### TUNEL assay

Dorsal skins were harvested, fixed in 4% PFA in PBS for 16 h at 4 °C, dehydrated in increasing sucrose concentrations (10%–15%–20%), embedded in Optimal Cutting Temperature (OCT) compound, frozen on liquid nitrogen, and cryosectioned (8–10 μm) (Cryostat Leica CM3050S). Sections were washed twice with PBS, fixed with 4% PFA for 10 min and again washed twice with PBST. Subsequently, sections were incubated with ProtK (10 µg/ml) for 5 min and washed twice with PBST. For Tunel staining, the “In situ Cell Death Detection Kit-TMR red” (Roche) kit was used according to the manufacturer’s instructions. Sections were washed 3 times in PBS and mounted with DAPI FluoroMount-G (Electron microscopy sciences BN17984-24).

### X-Gal staining

For X-Gal staining, dorsal skins were harvested, stretched on a membrane to keep the skin flat, embedded in OCT compound, immediately fresh frozen on liquid nitrogen and cryosectioned (20 µm). Sections were fixed for 10 min in 0.2% glutaraldehyde (Sigma Aldrich cat#G7776), washed three times for 5 min in PBS, and incubated with 1 mg/ml X-gal (Sigma Aldrich, cat#B4252) overnight at 37 °C. Sections were washed three times for 5 min in PBS and mounted with Immu-Mount mounting medium (Thermo scientific).

### Microscopy

Imaging of in situ hybridization, HE, and X-gal staining were performed with Zeiss upright AxioImagerM2 through a 20X objective with a tiling mode. Images of EdU labeling and Tunel assay were acquired with Zeiss LSM780 inverted confocal microscope through a 20X objective and with Zeiss upright AxioImagerM2 Apotome through a 20x objective with a tiling mode. Imaging of whole mount skin and hairs was performed with Zeiss stereomicroscope (Discovery.V12). Zen Black 11 (service pack 7) and Zen Blue 2.3 software were used to acquire all images that were processed with adobe Photoshop 24.5.0.

### Cell sorting

To sort and isolate DP cells, a whole skin was floated dermis side down on 0.25% Trypsin (GIBCO) at 4 °C overnight, minced, and stirred for 1 h in 0.2% collagenase at 37 °C. The cells were then sequentially filtered with 100 µM, 70 µM, and 40 µM strainers. YFP-positive cells were twice FACS-sorted on MoFlo Astrios (Backman Coulter): enrichment 1–2 mode was applied for the first sort and purify 1 mode was employed for the subsequent re-sort to achieve a purity of about 90% (Supplementary Fig. 11). FACS analyses were performed using Summit program.

### RNA sequencing and quality processing

Total RNA was purified from FACS-sorted cells utilizing the RNeasy Plus Micro kit (QIAGEN, #74034) according to manufacturer’s instructions. Integrity of the isolated RNA was tested using the Agilent RNA Pico Kit and Bioanalyzer of the “Genome Technology center” at the Faculty of Medicine Bar-Ilan university. Ribosomal depletion was performed on 100 ng of total RNA and libraries for Illumina sequencing were generated using the Nebnext Ultra Directional RNA kit (NEB, #E7420L). Quantification of the library was performed using dsDNA HS Assay Kit and QUBIT (Molecular Probes, Life Technologies). Additional qualitative and quantitative library measurements were performed by qPCR analysis using the illumina P5 and P7 primers. For optimal load on the sequencer, a standard library with similar characteristics was used. 2 nM of the library was denatured in 0.1 M NaOH for 5 min at room temperature, and 10pM was loaded onto the Flow Cell with 1% Phix library control. Libraries were sequenced on an Illumina HiSeq 2500 instrument, using a 50 cycles single-read sequencing mode.

### Bioinformatics for RNA-sequencing data

50 base single-end reads were trimmed and quality filtered using Trimmomatic-0.35^55^, and then mapped to the mouse genome (NCBI38/mm10) using Tophat (version 2)^56^. Mapped reads for each annotated ENSEMBL gene (GRCm38.p4) were counted using HTSeq (Version 0.6.1)^57^. Read count normalization and differential gene expression analysis was performed using DESeq2^58^.

### Statistical analysis

Statistical and graphical data analyses were performed using Microsoft Excel and Prism 7 (Graphpad) software. For All experiments at least three mice of each genotype were analyzed. To determine the statistical significance between two groups when normal distribution is observed, unpaired two-tailed Student’s *t*-test was employed. For the cell number analysis of the secondary germ and for the percentage of CycD1-positive DP cell analysis, Mann–Whitney test was used. The *P*-value and the statistical tests used were indicated in the figure legends.

### Reporting summary

Further information on research design is available in the Nature Portfolio Reporting Summary linked to this article.

### Supplementary information

Supplementary Information

Reporting Summary

### Source data

Source Data

## Data Availability

The RNA-seq dataset is available in the GEO repository under accession number GSE235480. Source data are provided with this paper.
