# Supplementary material for: Hdac1 and Hdac2 regulate the quiescent state and survival of hair-follicle mesenchymal niche
Source: Nat Commun. 2023 Aug 10;14:4820. doi: 10.1038/s41467-023-40573-7 (PMC10415406; doi:10.1038/s41467-023-40573-7)
Supplement: Supplementary file 1 — Supplementary Information [file 41467_2023_40573_MOESM1_ESM.pdf]

SUPPLEMENTARY INFORMATION

Hdac1 and Hdac2 regulate the quiescent state and survival of hair-follicle mesenchymal niche

Hadas Sibony-Benyamini[1], Emil Aamar[1], and David Enshell-Seijffers[1]

1 The Laboratory of Developmental Biology, The Azrieli Faculty of Medicine, Bar Ilan university, 8 Henrietta Szold, Safed, Israel

Correspondence to: david.enshell@biu.ac.il

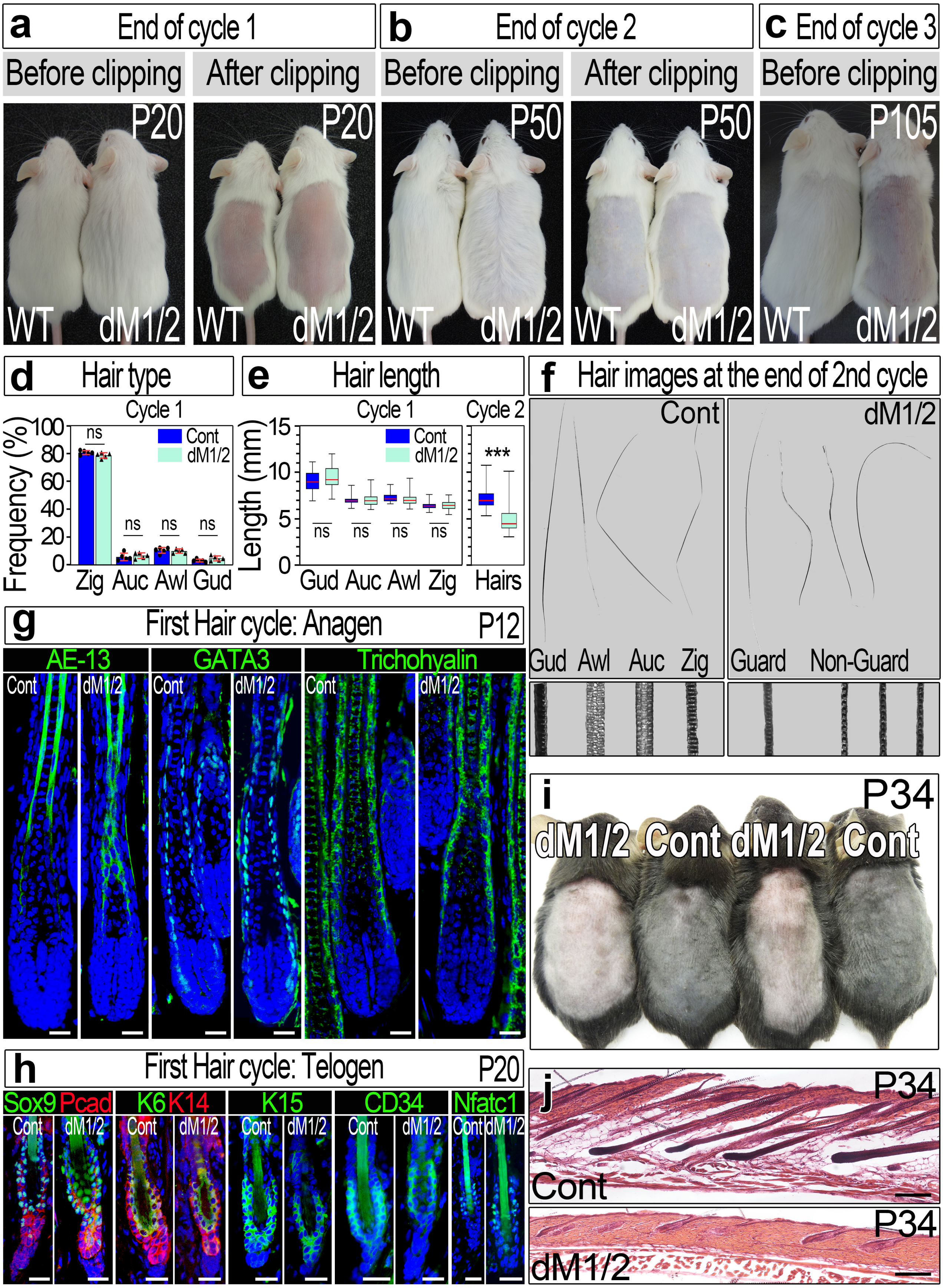

Supplementary Figure 1. Phenotypic analysis of the first and second hair cycles. (a-c) The same wild type (WT) and double mutant (dM1/2) pair of mice was followed through three subsequent hair cycles (first, second and third). At the end of each cycle, the hair coat was clipped, and pictures were taken before and after coat clipping. **(d)** Hairs were plucked from mid dorsal back skin at the end of first cycle, structurally analyzed and

scored for hair type. Mouse pelage is composed of four hair types; guard, awl, auchene and zigzag. All four hair types are clearly present and easily distinguished in the mutant. The hair type frequency is comparable between control and mutant mice. n=5 mice per genotype were analyzed and at least 200 hairs per mouse were scored. Data are mean ± SD. ns; not significant. **(e, f)** Hair length measurements and hair images are shown. At the end of the first cycle, hair length was measured and separately analyzed according to hair type (left in e). Hair length is comparable between control and mutant mice. n=5 mice per genotype were analyzed and at least 10 hairs per mouse per hair type were measured. At the end of the second cycle, hair structure is altered (f). Note that while guards can be readily identified in the mutant, auchenes, awls, and zigzags could not be reliably distinguished in the dM1/2 mutant and therefore were collectively designated non-guards (right image in f). Consequently, hair length measurements of all hair types were collectively analyzed without subclassifying into hair type (right in e). Hair length of mutant mice is shorter than controls. n=5 mice per genotype were analyzed and at least 40 hairs per mouse were measured. Data are presented by box-and-whisker plots (red midline, median; box, 25th and 75th percentiles; whiskers, minimum and maximum). ns; not significant, *** P=1.24E-06, unpaired two-tailed Student's t test. **(g)** Immunostaining for hair keratins (AE-13), GATA3 and Trichohyalin during mid anagen of the first cycle is shown. Hair keratins are expressed specifically in the hair cortex and Trichohyalin is expressed in both the hair medulla and IRS. Note that the levels and pattern of these differentiation markers remain unaltered in mutant mice. GATA3 expression is specific to the IRS, and GATA3 transcriptional activity is required for IRS formation. The expression levels and pattern of this master regulator are comparable between control and mutant mice. Together these data suggest that differentiation programs during anagen of the first cycle occur normally in the mutant. n=4 mice per genotype. Scale bar; 20µm. **(h)** Immunostaining for structural markers (Pcad, K14, K6), stem cell markers (CD34, K15) and transcription factors that play important role in bulge stem cell activity (Sox9, Nfatc1) during the telogen of the first cycle revealed no alterations in stem cell properties and the overall structure of the telogen hair follicle. n≥3 mice per genotype. Scale bar; 20µm. **(i-j)** Ablation of Hdac1 and Hdac2 in the DP shortens the duration of the anagen phase of the second hair cycle. Pigment production and deposition in mouse skin are restricted to the hair follicle and hair shaft respectively, and occurs only during the anagen phase when melanocytes in the bulb region produce and transfer their pigment to differentiating matrix cells. Consequently, when the hair coat is clipped during anagen or telogen, the skin appears black or white/pink respectively. Therefore, skin color can be used as an indicator for the phase of the hair cycle. In i, a representative example with two control and two double mutant littermates at P34 is shown, illustrating that while the control mice are still in late anagen, the double mutants appear to be in the telogen phase. In j, HE staining of skin sections obtained from control (upper panel) and double mutant (lower panel) mice at P34 illustrates that all follicles in the control are in the anagen phase while in the double mutant all follicles have already entered the telogen phase. n=10 for control mice and n=15 for double mutant mice. Scale bar; 200µm. Source data are provided as a Source Data file.

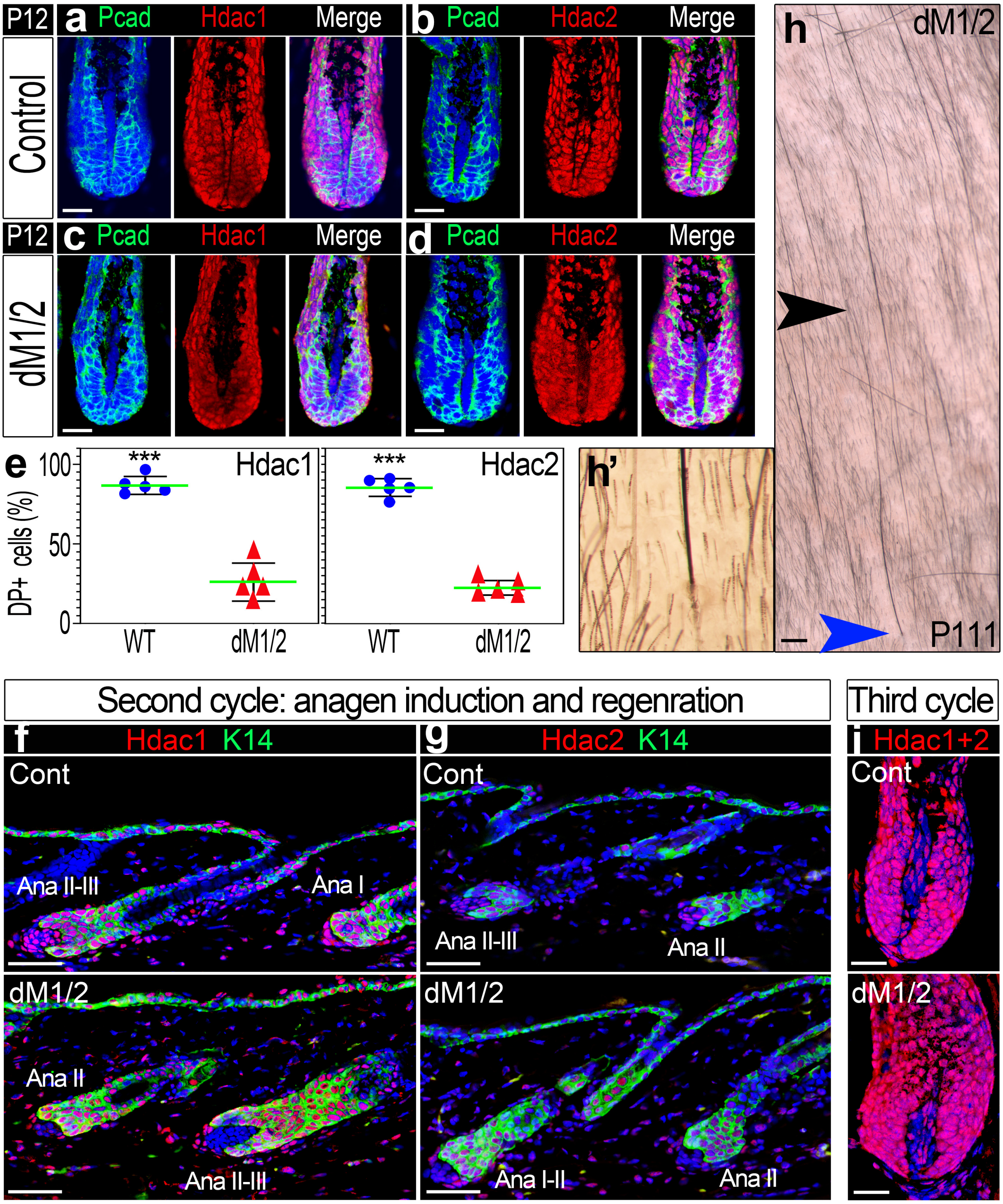

Supplementary Figure 2. Deletion of Hdac1 and Hdac2 in the DP is largely efficient. (a-d) Immunostaining for Hdac1 (a, c) and Hdac2 (b, d) during mid anagen (P12) of the first hair cycle reveals efficient deletion specifically in the DP of the dM1/2 mutant already at P12. Pcad is also included to outline the DP. In each case, the same follicle is shown on the left, middle and right panels. On the left, Pcad and DAPI (blue) co-immunostaining is shown. On the middle, only Hdac immunostaining is exhibited, and on the right, a merge is depicted. Scale bar; 20μm. **(e)** Quantification of Hdac1 and Hdac2 expression in DP cells at P12 is shown. Hdac-positive and Pcad-negative cells were scored as DP-positive cells (DP+). Note that the ratio between control and dM1/2 mutant mice indicates deletion efficiency of about 70-80%. Data are mean ±SD. 5 mice were analyzed and 81-100 hair follicles per mouse were scored. ***; P=6.86E-06, unpaired two-

tailed Student's t test. **(f, g)** Regeneration during the second cycle is normal despite the absence of Hdac1 and Hdac2 in the DP. Double immunostaining for K14 (Green) and Hdac1 (red) or for K14 and Hdac2 are shown, respectively. The specific regeneration stage for each follicle is indicated. Nuclei are in blue (DAPI). 4 controls and 3 dM1/2 mutants were analyzed. Scale bar; 50µm. **(h, i)** Single isolated dM1/2 mutant follicles with inefficient Hdac1/2 deletion in the DP are capable to induce anagen of the third cycle. In h, dorsal view of a dM1/2 mutant mouse is shown to illustrate the presence of isolated and unclipped hairs that occasionally grow during the third cycle. The black and blue arrowheads indicate the tip and the base of a growing hair, respectively. Scale bar; 500µm. In h', higher magnification of the field indicated by the blue arrowhead in h is displayed to demonstrate the emergence point of the growing hair and the surrounded clipped hairs of telogen follicles. In i, co-immunostaining for Hdac1 and Hdac2 (both red) of control (upper panel) and dM1/2 mutant (lower panel) follicle during the third cycle is shown. Nuclei are in blue (DAPI). Scale bar; 20µm. For each genotype, 5 mice were analyzed. Source data are provided as a Source Data file.

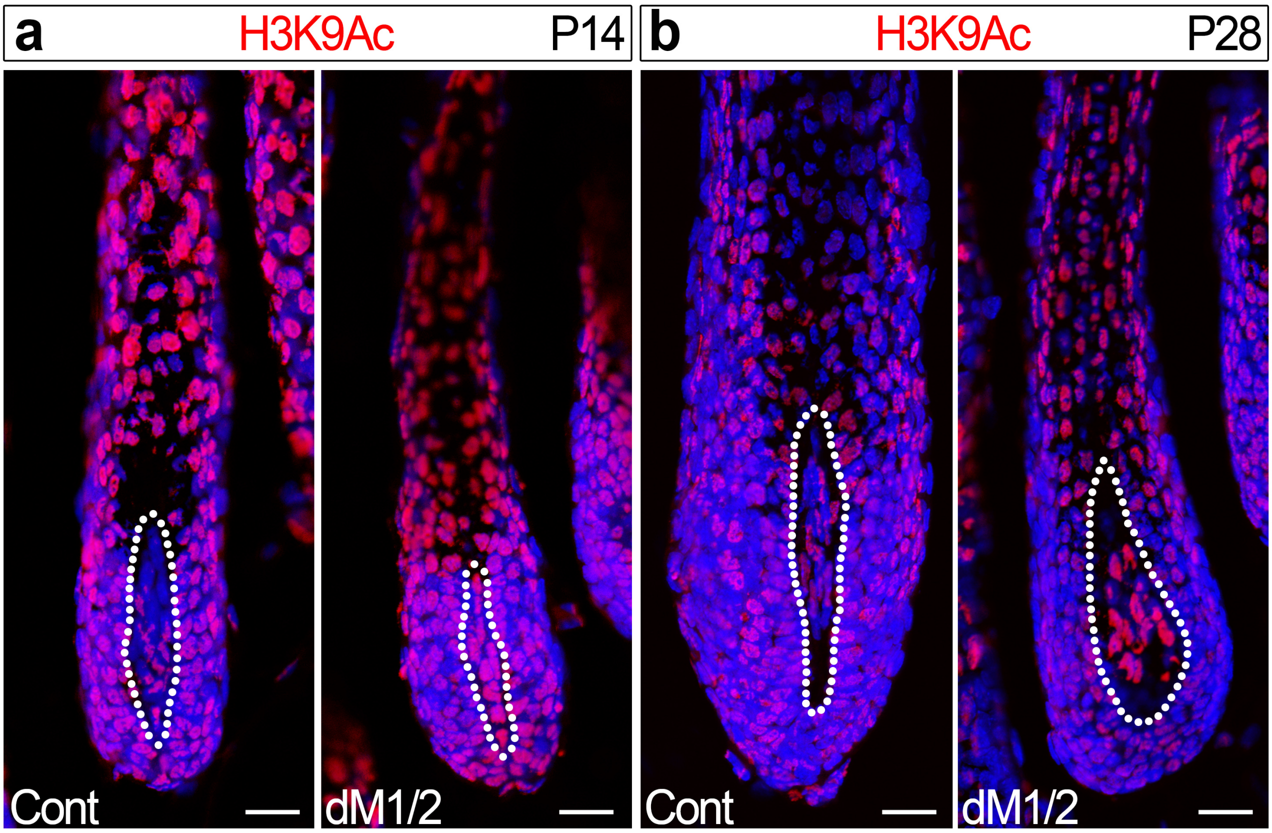

Supplementary Figure 3. Ablation of Hdac1 and Hdac2 in the DP results in chromatin over-acetylation. Immunostaining for H3K9Ac (Red) reveals increased H3K9Ac levels specifically in the DP of the dM1/2 mutant as compared to control. **(a)** Mid to late anagen of the first cycle (P14). **(b)** Mid anagen of the second cycle (P28). The white dotted line demarcates the DP. Nuclei are counterstained with DAPI (Blue). For each genotype, 3 mice were analyzed. Scale bar; 20µm.

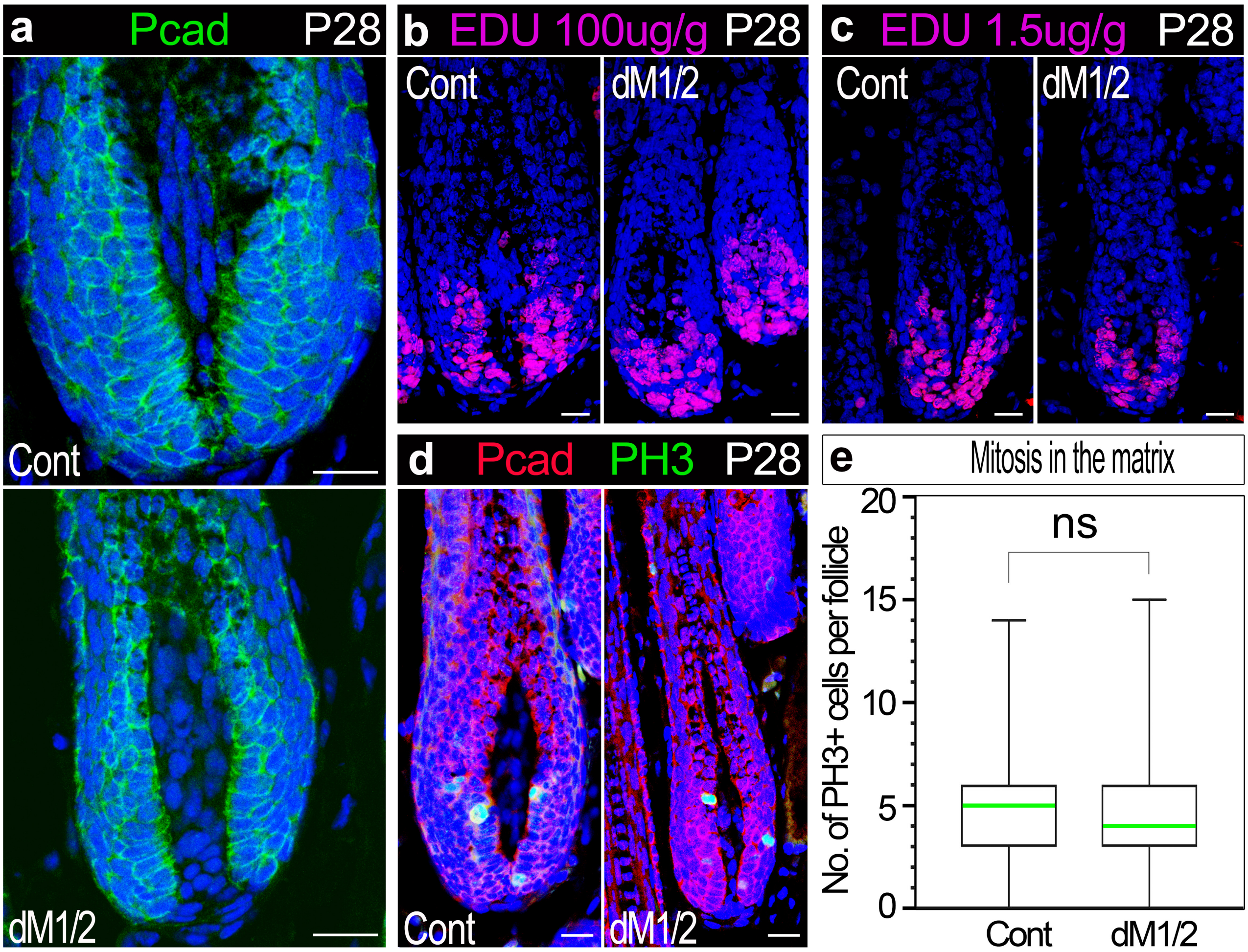

Supplementary Figure 4. The bulb size is reduced while the proliferation rate of matrix cells remains unaltered in the absence of Hdac1 and Hdac2 in the DP. (a) Pcad immunostaining of skin sections from control (upper panel) and dM1/2 mutant (lower panel) mice at P28 is shown to illustrate the bulb morphology and to distinguish between the matrix and the DP. This is a higher magnification of the image displayed in Fig. 1i. **(b, c)** Control and dM1/2 mutant mice were injected with Edu at P28 and chased for 15-30 min. In b, standard dosage of 100ug/gr Edu and a chase of 30 min were used. In c, very low dosage of 1.5ug/gr Edu and a chase of 15 min were employed. n=3 mice per genotype. **(d)** Double immunostaining for phospho-Histone 3 (pH3) (Green) and Pcad (Red) is used to label mitotic cells in the matrix. **(e)** Quantification of the number of mitotic cells (based on pH3-positive cells) in the matrix per follicle per section is shown. Data are presented by box-and-whisker plot (green midline, median; box, 25th and 75th percentiles; whiskers, minimum and maximum). n=5 mice were analyzed per genotype, and 85-100 hair follicles per mouse were scored. ns; not significant, unpaired two-tailed Student's t test. Scale bar in all panels; 20μm. Source data are provided as a Source Data file.

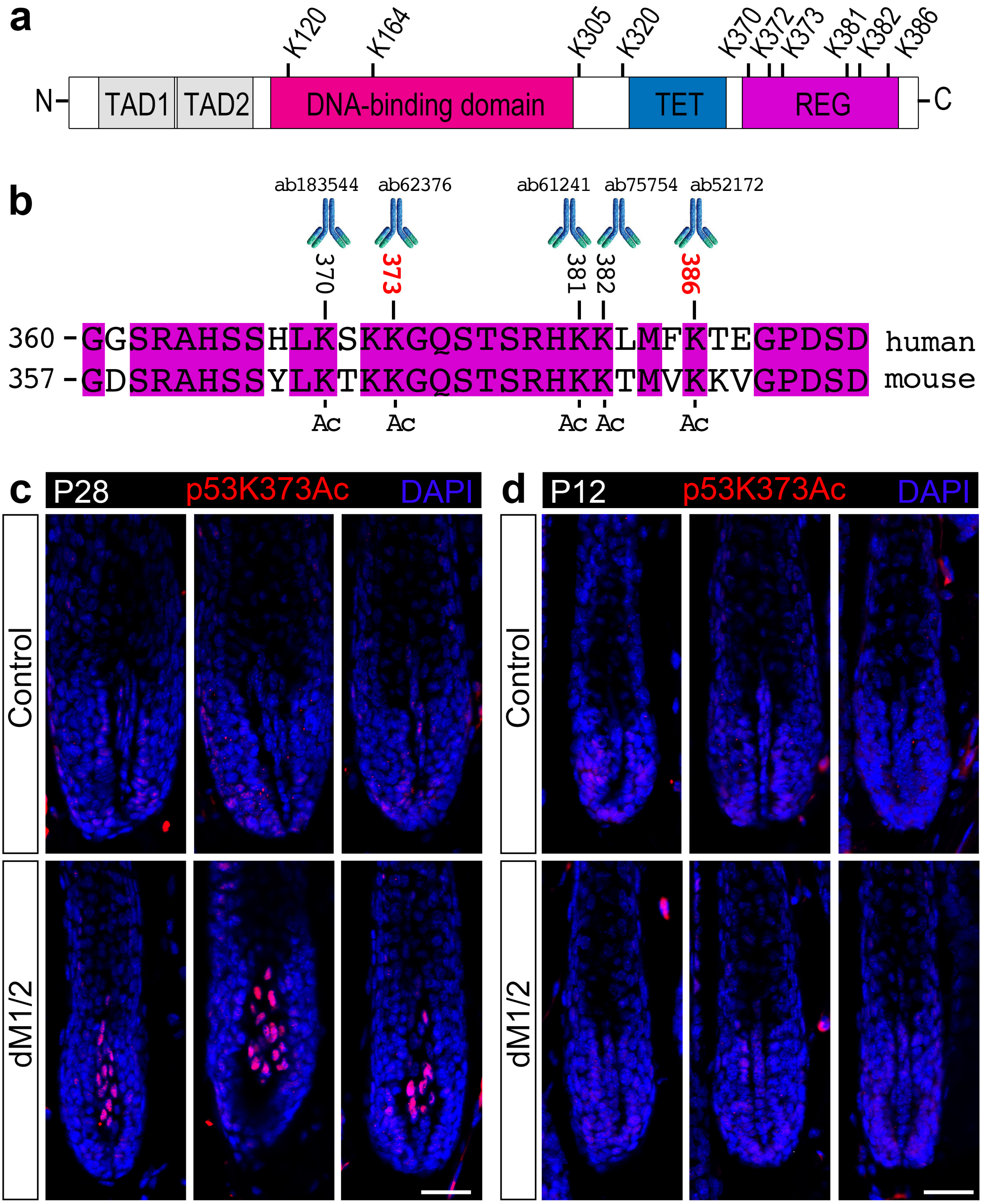

Supplementary Figure 5. p53 in the DP is acetylated in the absence of Hdac1/2 only during the second cycle. (a) Schematic of p53 is shown to illustrate the p53 domain organization and to highlight lysine residues known to be acetylated by acetyltransferases. TAD; transactivation domain, TET; tetramerization domain, REG; regulatory domain. Note that the acetylation of the lysine residues in the REG domain promotes the open conformation of p53 by inhibiting the ability of its REG domain to bind and occlude the DNA-binding domain. **(b)** Commercial antibodies that are tested in the current study and their recognized acetylated lysine residues are presented. The relevant amino acid sequences of the human and mouse REG domain are shown and aligned. Note that all commercial antibodies raised against the human sequence. Identical residues are

highlighted in magenta. Only two antibodies demonstrate reactivity in immunostaining (highlighted in red). **(c)** Immunostaining of skin sections from P28 control and dM1/2 mutant mice with anti-p53K373Ac (red) is shown to demonstrate p53 acetylation only in the DP of dM1/2 mutant during the second cycle. Three representative examples per genotype are depicted. DAPI (blue) is used to label nuclei. Scale bar; 50µm. **(d)** Immunostaining of skin sections from P12 control and dM1/2 mutant mice with anti-p53K373Ac (red) is shown to illustrate the lack of p53 acetylation during the first cycle regardless of the genotype. DAPI (blue) is used to label nuclei. n=3 mice per genotype. Scale bar; 50µm.

---

a First-cycle TUNEL labeling

P12	Control #1	Control #2	Control #3	Control Average	dM1/2 #1	dM1/2 #2	dM1/2 #3	dM1/2 Average
HF with 0 DP/Tunel+ Cells	100 %	100 %	100 %	**100 %**	100 %	100 %	100 %	**100 %**
HF with ≥1 DP/Tunel+ Cells	0%	0%	0 %	**0 %**	0 %	0 %	0 %	**0 %**
Total number of HF	77	78	99	**254**	82	70	78	**230**

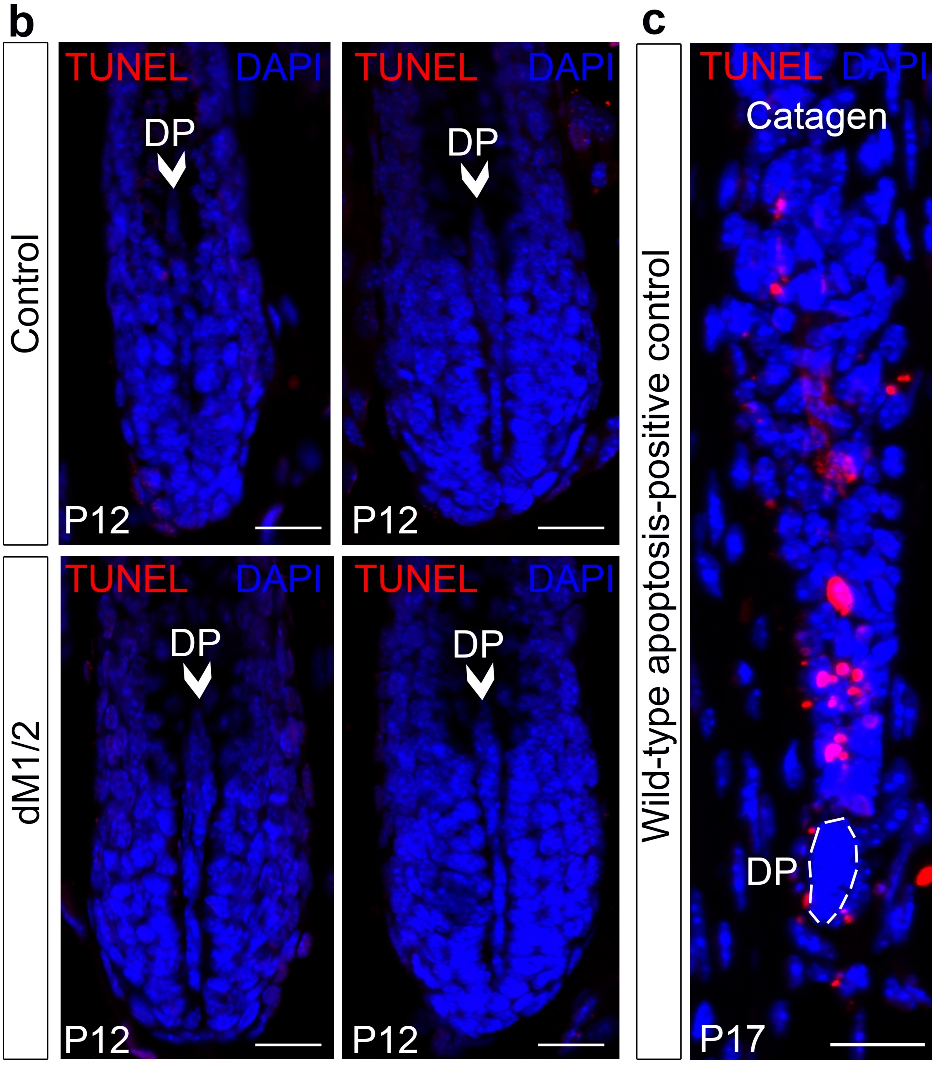

Supplementary Figure 6. DP survival of dM1/2 mutant mice during the first cycle is normal. (a) Tunel staining of P12 control and dM1/2 mutant mice was used to score apoptosis in the DP. Three mice per genotype were analyzed and the total number of follicles is indicated. HF; hair follicle. Ave; average. **(b)** Tunel staining (red) of two representative follicles per genotype is shown. White arrow heads indicate the most distal point of the DP. DAPI (blue) labels nuclei. Scale bar; 20µm. **(c)** Catagen follicles from a wild-type mouse were used to positively control the Tunel staining (red). A representative example is shown. White broken line demarcates the DP. DAPI (blue) labels nuclei. n=3 mice per genotype. Scale bar; 20µm.

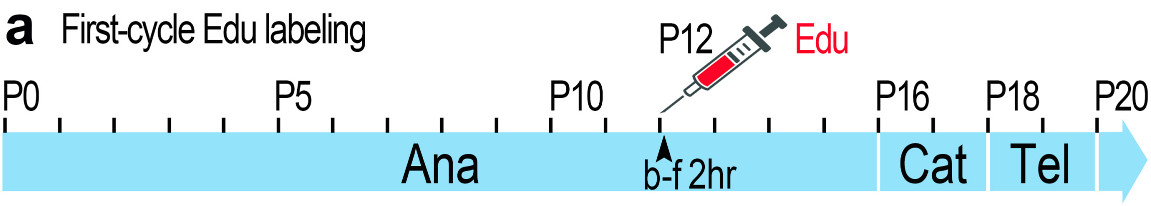

b P12	Control #1	Control #2	Control #3	Control Average	dM1/2 #1	dM1/2 #2	dM1/2 #3	dM1/2 Average
HF with 0 DP/Edu+ Cells	100%	100%	98.8%	99.6%	100%	98.8%	100%	99.6%
HF with ≥1 DP/Edu+ Cells	0%	0%	1.2%	0.4%	0%	1.2%	0%	0.4%
Total number of HF	105	81	80	266	77	84	60	221

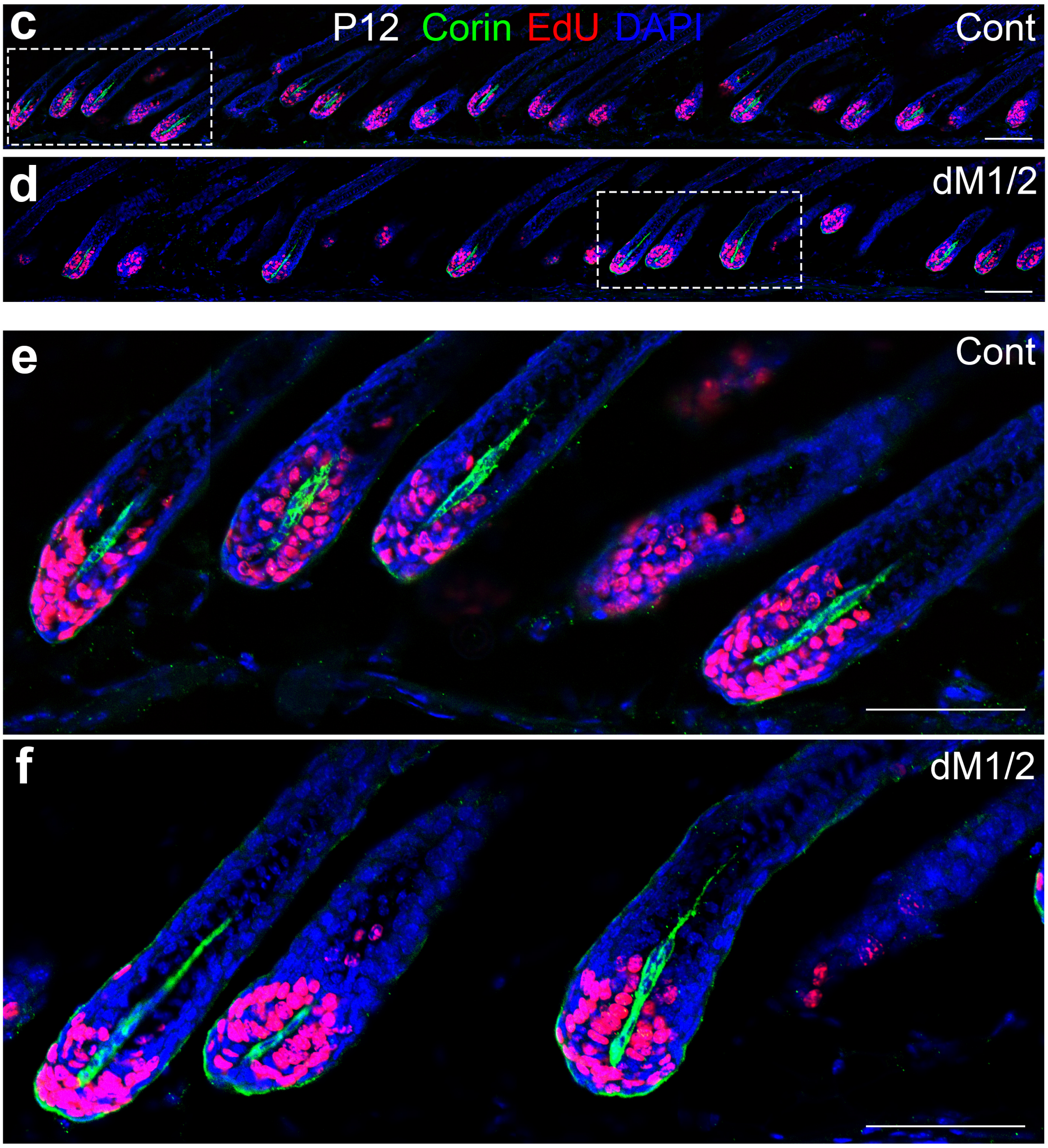

Supplementary Figure 7. DP dormancy in dM1/2 mutant mice is maintained during the first cycle. (a) Edu labeling experiment during the first cycle is schematically shown. Edu administration was conducted at

P12, and Edu incorporation was tested two hours later. On top, a time scale of postnatal day (P) of the first cycle is shown. The black arrowhead indicates time of skin harvest, and the corresponding image is indicated beneath the arrowhead. **(b)** Edu labeling of P12 control and dM1/2 mutant mice was used to score proliferation in the DP. Three mice per genotype were analyzed and the total number of follicles is indicated. HF; hair follicle. **(c, d)** Representative confocal images from control and dM1/2 mutant mice are depicted to illustrate the abundance of Edu labeling (red) in the matrix and the lack of Edu labeling in the DP regardless of the genotype. Immunostaining for Corin (green) was also included to specifically mark the DP. DAPI (blue) labels nuclei. n=3 mice per genotype. Scale bar; 100µm. **(e, f)** Higher magnifications of the fields demarcated in white dash rectangles in c and d. Scale bar; 100µm.

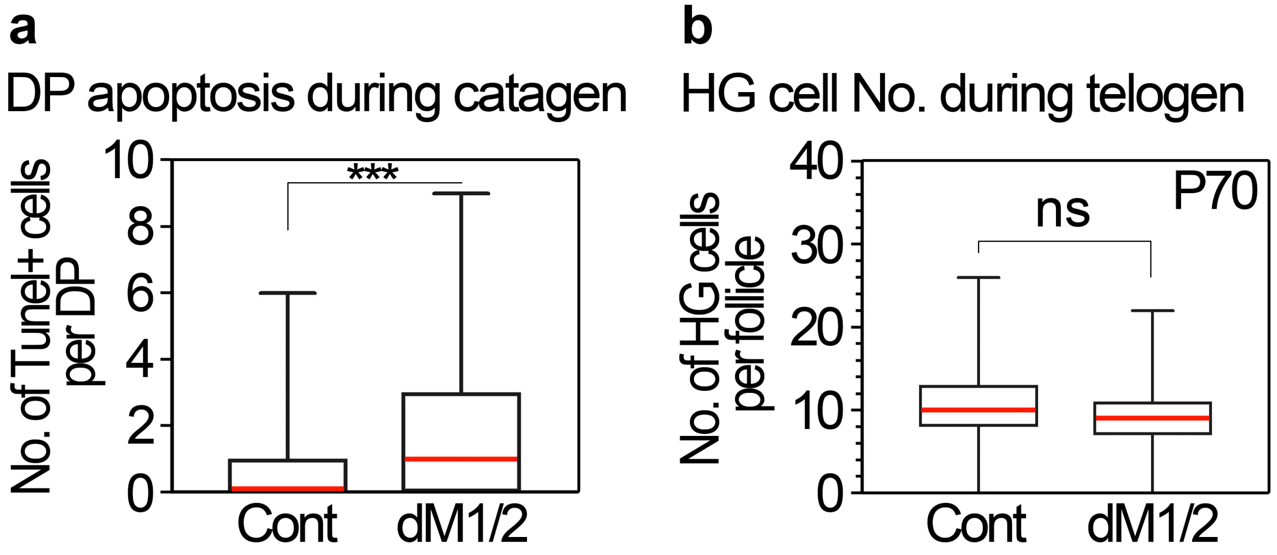

Supplementary Figure 8. DP apoptosis during catagen does not affect the formation of secondary hair germ. (a) The number of Tunel+ cells per DP per follicle during catagen is shown. Data are presented by box-and-whisker plot (red midline, median; box, 25th and 75th percentiles; whiskers, minimum and maximum). n=5 mice per genotype and 50-100 follicles per mouse were scored. *** P<0.0001, two-sided Mann-Whitney test. **(b)** Pcad immunostaining was used to score for the number of secondary hair germ (HG) cells per follicle. Data are presented by box-and-whisker plots (red midline, median; box, 25th and 75th percentiles; whiskers, minimum and maximum). n=3 mice per genotype and ~100 follicles per mouse were scored. ns; not significant, two-sided Mann-Whitney test. Source data are provided as a Source Data file.

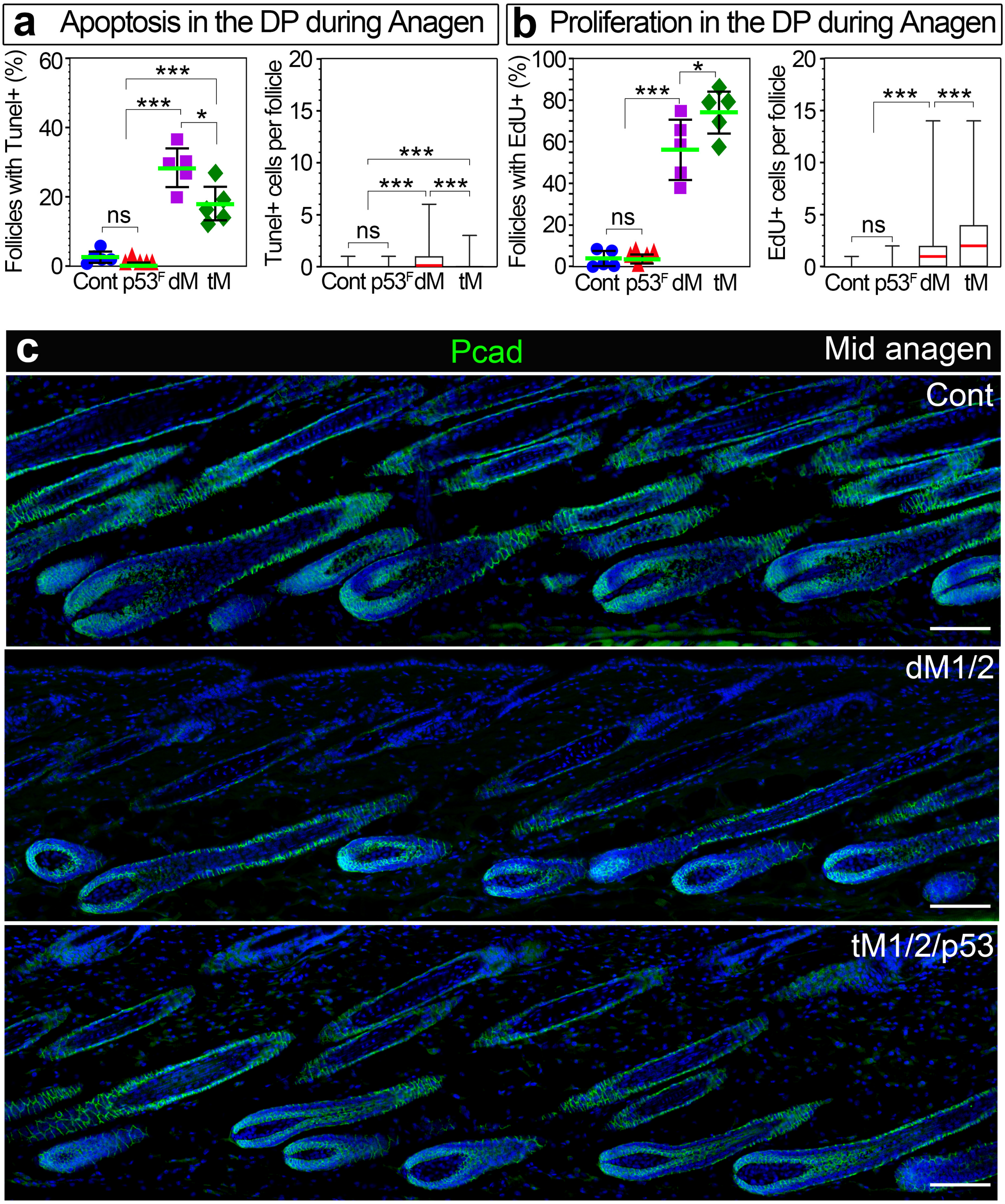

Supplementary Figure 9. p53 activity in the DP restrains proliferation and promotes apoptosis in a Hdac1/2-dependent mode. (a) Quantification of apoptotic cells in the DP during anagen. On the left, the number of follicles per mouse (in percentage) with Tunel-positive cells in the DP is presented. Data are mean ± SD. ns; not significant, * P=0.01, *** P=0.0001, unpaired two-tailed Student's t test. On the right, the number of Tunel-positive cells in the DP per follicle is displayed. Data are presented by box-and-whisker plots (red midline, median; box, 25th and 75th percentiles; whiskers, minimum and maximum). ***P<0.0001, unpaired two-tailed Student's t test. Cont; control (n=5 mice), $p53^F$; homozygote for the floxed allele of p53 (n=5 mice), dM; double mutant for Hdac1 and Hdac2 (n=5 mice), tM; triple mutant for Hdac1, Hdac2 and p53 conditional knockout (n=5 mice). 52-104 follicles per mouse were scored. **(b)** Quantification of proliferating cells in the

DP during anagen by EdU incorporation. On the left, data are mean ± SD. ns; not significant, * P=0.048, *** P=000.1, unpaired two-tailed Student's t test. On the right data are presented by box-and-whisker plots (red midline, median; box, 25th and 75th percentiles; whiskers, minimum and maximum). ***P<0.0001, unpaired two-tailed Student's t test. n=5 mice per genotype and 42-103 follicles per mouse were scored. **(c)** Immunostaining for Pcad during the anagen of the second hair cycle is shown to outline the DP and to demonstrate the extremely large DP in the triple mutant. n=5 mice per genotype. Scale bar; 100µm. Source data are provided as a Source Data file.

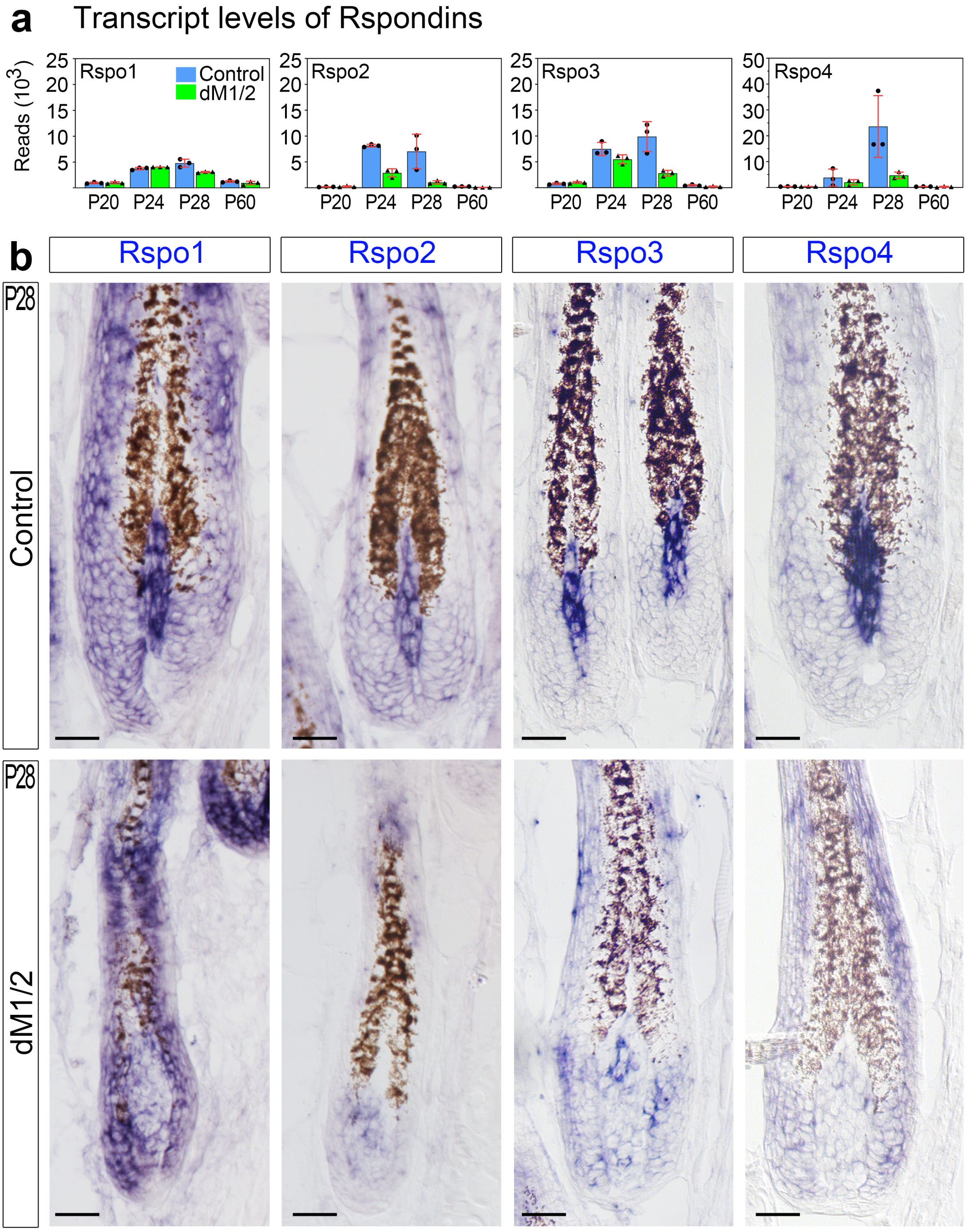

Supplementary Figure 10. The expression of Rspondins is down regulated in the DP of the dM1/2 mutant. (a) The number of normalized reads from the RNA-seq analysis for all 4 Rspondins is shown. Data

are mean ± SD. **(b)** *In situ* hybridization for all 4 Rspondins (blue) is presented. n=3 mice per genotype were analyzed. Scale bar; 25μm. Source data are provided as a Source Data file.

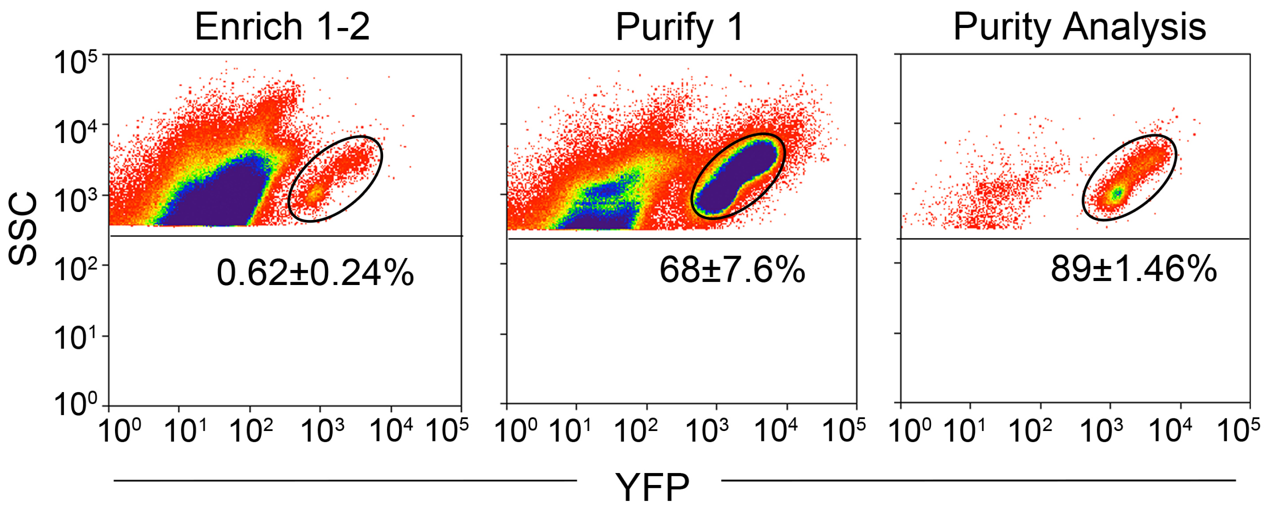

Supplementary Figure 11. FACS sorting strategy to isolate DP cells. Scatter plots of FACS sorting are shown. Back skins were harvested and individually dissociated into a single-cell suspension. For each mouse skin, YFP-positive cells were sorted twice. The plot on the left represents the first sort to enrich for YFP-positive cells using the Enrich 1-2 mode, the middle shows the re-sort of the YFP enriched cells using the Purify 1 mode, and the plot on the right displays the purity analysis after two sorts. n=3 mice per genotype per stage. Data are mean ± SD.
