# Supplementary material for: Hdac1 and Hdac2 regulate the quiescent state and survival of hair-follicle mesenchymal niche
Source: Nat Commun. 2023 Aug 10;14:4820. doi: 10.1038/s41467-023-40573-7 (PMC10415406; doi:10.1038/s41467-023-40573-7)
Supplement: Supplementary file 2 — Reporting Summary [file 41467_2023_40573_MOESM2_ESM.pdf]

nature portfolio

Corresponding author(s): David Enshell-Seijffers

Last updated by author(s): Jul 1, 2023

Reporting Summary

Nature Portfolio wishes to improve the reproducibility of the work that we publish. This form provides structure for consistency and transparency in reporting. For further information on Nature Portfolio policies, see our Editorial Policies and the Editorial Policy Checklist.

Statistics

For all statistical analyses, confirm that the following items are present in the figure legend, table legend, main text, or Methods section.

n/a	Confirmed	
☐	☒	The exact sample size (*n*) for each experimental group/condition, given as a discrete number and unit of measurement
☐	☒	A statement on whether measurements were taken from distinct samples or whether the same sample was measured repeatedly
☐	☒	The statistical test(s) used AND whether they are one- or two-sided<br>*Only common tests should be described solely by name; describe more complex techniques in the Methods section.*
☐	☒	A description of all covariates tested
☐	☒	A description of any assumptions or corrections, such as tests of normality and adjustment for multiple comparisons
☐	☒	A full description of the statistical parameters including central tendency (e.g. means) or other basic estimates (e.g. regression coefficient) AND variation (e.g. standard deviation) or associated estimates of uncertainty (e.g. confidence intervals)
☐	☒	For null hypothesis testing, the test statistic (e.g. F, t, r) with confidence intervals, effect sizes, degrees of freedom and P value noted<br>*Give P values as exact values whenever suitable.*
☒	☐	For Bayesian analysis, information on the choice of priors and Markov chain Monte Carlo settings
☐	☒	For hierarchical and complex designs, identification of the appropriate level for tests and full reporting of outcomes
☒	☐	Estimates of effect sizes (e.g. Cohen's *d*, Pearson's *r*), indicating how they were calculated

Our web collection on statistics for biologists contains articles on many of the points above.

Software and code

Policy information about availability of computer code

Data collection	Microscopy Images were acquired using Zen Black 11 (service pack 7) and Zen Blue 2.3 software, and finalized using adobe Photoshop 24.5.0.
Data analysis	RNASeq data Bioinformatic analysis was performed with Trimmomatic-0.35 (Bolger et al., 2014), Tophat (version 2) (Kim et al., 2013), HTSeq (Version 0.6.1) (Anders et al., 2015), DESeq2 (Love et al., 2014). For statistical analysis, Microsoft Excel and Prism 7 (Graphpad) were used.

For manuscripts utilizing custom algorithms or software that are central to the research but not yet described in published literature, software must be made available to editors and reviewers. We strongly encourage code deposition in a community repository (e.g. GitHub). See the Nature Portfolio guidelines for submitting code & software for further information.

Data

Policy information about availability of data

All manuscripts must include a data availability statement. This statement should provide the following information, where applicable:

- Accession codes, unique identifiers, or web links for publicly available datasets
- A description of any restrictions on data availability
- For clinical datasets or third party data, please ensure that the statement adheres to our policy

The RNA-seq dataset is available in the GEO repository (GSE235480). Source data are provided with this paper.

Research involving human participants, their data, or biological material

Policy information about studies with human participants or human data. See also policy information about sex, gender (identity/presentation), and sexual orientation and race, ethnicity and racism.

Reporting on sex and gender	N/A
Reporting on race, ethnicity, or other socially relevant groupings	N/A
Population characteristics	N/A
Recruitment	N/A
Ethics oversight	N/A

Note that full information on the approval of the study protocol must also be provided in the manuscript.

Field-specific reporting

Please select the one below that is the best fit for your research. If you are not sure, read the appropriate sections before making your selection.

☒ Life sciences ☐ Behavioural & social sciences ☐ Ecological, evolutionary & environmental sciences

For a reference copy of the document with all sections, see nature.com/documents/nr-reporting-summary-flat.pdf

Life sciences study design

All studies must disclose on these points even when the disclosure is negative.

Sample size	For all experiments, at least 3 mice per genotype were used, which is a common biological replicates in the field, and at least a toatal of 300 follicles were scored.
Data exclusions	No data were excluded from the analysis.
Replication	Reproducibility was based on at least 3 biological replicates.
Randomization	Control and mutant mice were selected based on their genotype.
Blinding	Blinding was not used since control and mutant mice are readily distinguished based on their phenotypes.

Reporting for specific materials, systems and methods

We require information from authors about some types of materials, experimental systems and methods used in many studies. Here, indicate whether each material, system or method listed is relevant to your study. If you are not sure if a list item applies to your research, read the appropriate section before selecting a response.

Materials & experimental systems

n/a	Involved in the study	
☐	☒	Antibodies
☒	☐	Eukaryotic cell lines
☒	☐	Palaeontology and archaeology
☐	☒	Animals and other organisms
☒	☐	Clinical data
☒	☐	Dual use research of concern
☒	☐	Plants

Methods

n/a	Involved in the study	
☒	☐	ChIP-seq
☐	☒	Flow cytometry
☒	☐	MRI-based neuroimaging

Antibodies

Antibodies used	Primary antibodies (star indicates that antigen retrieval is required): Guinea pig polyclonal anti-K14 (1:500, Acris #BP-5009), Rabbit polyclonal anti-HDAC1 (1:200*, Abcam #ab19845), Rabbit monoclonal anti-HDAC2 [clone Y461] (1:500, Abcam #ab32117), Rabbit polyclonal anti-H3K9Ac (1:1000, Abcam #ab10812), Rabbit polyclonal anti-p53K386Ac (1:200, Abcam # ab52172), Rabbit monoclonal

anti-p53K370Ac [clone EPR1749] (1:100, Abcam #ab183544), Rabbit monoclonal anti-p53K373Ac [clone EP356(2)AY] (1:3200*, Abcam #ab62376), Rabbit polyclonal anti-p53K381Ac (1:100, Abcam #ab61241), Rabbit monoclonal anti-p53K382Ac [clone EPR358(2)] (1:100, Abcam #ab75754), Rabbit polyclonal anti-Corin (1:800, Enshell-Seijffers et al. 2010), Rabbit monoclonal anti-Cycin D1 [clone 92G2] (1:100*, Cell signaling #29785), Mouse monoclonal anti-hair cortex Cytokeratin [clone AE13] (1:100*, Abcam #ab16113), Rabbit polyclonal anti-Trichohyalin (1:100*, Santa cruz # sc-98968), Mouse monoclonal anti-GATA3 [clone HG3-31] (1:200*, Santa cruz #sc-268), Rat monoclonal FITC-conjugated anti-CD34 [clone RAM34] (1:50*, eBioscience #11-0341), Rabbit polyclonal anti-5OX9 (1:250*, Millipore #AB5535), Rat monoclonal anti-P-Cadherin (Pcad) [clone 106020] (1:100*, BD #MAB761), Rabbit polyclonal anti-K6 (1:500*, Covance #PRB-169P), Mouse monoclonal anti- Nfatcl [clone 7A6] (1:50*, Santa cruz #sc-7294, 7A6), Mouse monoclonal anti-K15 [clone LHK15] (1:100*, Thermo scientific #M5-1068), Rabbit polyclonal anti-Phospho-Histone 3 (1:250, Abcam #ab 5176).
Secondary antibodies: Donkey anti Guinea pig-Cy5, (1:500, Jackson immunoresearch #706-175-148), Donkey anti Rabbit-TRITC, (1:1000, Jackson immunoresearch #711-025-152), Donkey anti Rabbit-FITC, (1:1000, Jackson immunoresearch, #711-095-152), Donkey anti mouse-Alexa Fluor 488, (1:1000, Jackson immunoresearch, #715-545-150), Donkey anti Rat-Cy5, (1:1000, Jackson immunoresearch, #712-175-753)

Validation

All antibodies were validated by western blot analysis described in the manufacturers' website.

Animals and other research organisms

Policy information about studies involving animals; ARRIVE guidelines recommended for reporting animal research, and Sex and Gender in Research

Laboratory animals

The CMV-cre transgenic mouse line, the p53 conditional knockout line, the reporter mouse lines ROSA26 tdTomato and ROSA26 EYFP, and the Axin2-lacZ Wnt reporter line were obtained from Jackson Laboratory. Hdac1 and Hdac2 conditional knockout mice were kindly provided by Eric Olson (The University of Texas Southwestern Medical Center). The DP-specific Corin-cre mouse line was previously generated and kindly provided by Bruce Morgan (Harvard medical school). To establish the p53 knockout line, the p53-floxed mouse line was crossed with the CMV-cre line that ubiquitously expresses the cre recombinase in all cells. Progeny were outcrossed to exclude the CMV-cre allele. The age of mice used in this study was between postnatal day 12 (P12) and P105.

Wild animals

The study did not involve wild animals.

Reporting on sex

Telogen of the first hair cycle in males lasts for only two days while in females continues longer and varies. When the telogen of the first cycle was analyzed, dorsal skins from P20 females were collected to avoid stage unambiguity. When the anagen of the second cycle was analyzed, only males were used to exploit the synchronized nature of their anagen induction and thus minimize hair cycle variation. The telogen of the second cycle lasts for weeks, and thus both males and females were analyzed.

Field-collected samples

The study did not involve samples collected from the field.

Ethics oversight

The Institutional Animal Care and Use Committee of Bar Ilan University.

Note that full information on the approval of the study protocol must also be provided in the manuscript.

Flow Cytometry

Plots

Confirm that:

- [x] The axis labels state the marker and fluorochrome used (e.g. CD4-FITC).
- [x] The axis scales are clearly visible. Include numbers along axes only for bottom left plot of group (a 'group' is an analysis of identical markers).
- [x] All plots are contour plots with outliers or pseudocolor plots.
- [x] A numerical value for number of cells or percentage (with statistics) is provided.

Methodology

Sample preparation

To sort and isolate DP cells, a whole skin was floated dermis side down on 0.25% Trypsin (GIBCO) at 4°C overnight, minced, and stirred for 1hr in 0.2% collagenase at 37°C. The cells were then sequentially filtered with 100µM, 70µM and 40µM strainers.

Instrument

MoFlo Astrios, Backman Coulter

Software

Summit program

Cell population abundance

YFP-positive cells were twice FACS-sorted on MoFlo Astrios (Backman Coulter): enrichment 1–2 mode was applied for the first sort and purify 1 mode was employed for the subsequent re-sort to achieve a purity of about 90%.

Gating strategy

The Y-axis represents SSC and the X-axis represents YFP positive cells. Since FACS sorting relies on endogenous expression of YFP in the DP, the YFP-positive population is determined based on single cell preparation of a skin derived from wild type mice that are unable to express YFP.

☒ Tick this box to confirm that a figure exemplifying the gating strategy is provided in the Supplementary Information.
